# Standardized microhaplotype databases and frameworks for assessing and mining crop genetic diversity

**DOI:** 10.1007/s00122-026-05340-4

**Published:** 2026-08-25

**Authors:** Dongyan Zhao, Meng Lin, Cristiane H. Taniguti, Alexander M. Sandercock, Shufen Chen, Ebrahiem Babiker, Nahla V. Bassil, E. Charles Brummer, Jose Roberto Camacho, Warren Chatwin, Shu-Yun Chen, Shaun J. Clare, Guilherme da Silva Pereira, Maria David, Simon Phillip Fraher, Michael Hardigan, Angelyn Hilton, Lillian M. Hislop, Brian M. Irish, Moctar Kante, Tae Hwa Kim, Chong-Wei Lee, Hannele Lindqvist-Kreuze, Jenyne Loarca, Po-Hsien Lu, Cesar Agusto Medina Culma, Jose Fabián Jiménez Morales, James Polashock, John H. Price, Heathcliffe Riday, Deborah A. Samac, Devinder Sandhu, Reuben Ssali, Ruth Mayela Castro Vásquez, Phillip A. Wadl, Xinwang Wang, Seymour A. Webster, Zhanyou Xu, G. Craig Yencho, Craig. T. Beil, Moira J. Sheehan

**Affiliations:** 1 Breeding Insight, University of Florida, Gainesville, FL 32611 USA; 2 USDA ARS Corn Host Resistance Research Unit, Mississippi State, MS 39762 USA; 3 USDA ARS National Clonal Germplasm Repository, Corvallis, OR 97333 USA; 4 Department of Plant Sciences, University of California Davis, Davis, CA 95616 USA; 5 The Instituto Nacional de Innovación y Transferencia en Tecnología Agropecuaria (INTA), San José, Costa Rica; 6 USDA ARS Pecan Breeding and Genetics Program, College Station, TX 77845 USA; 7 Department of Agronomy, College of Agriculture and Natural Resources, National Chung Hsing University, Taichung, Taiwan; 8 Carlsberg Research Laboratory, J. C. Jacobsens Gade 4, 1799 Copenhagen V, Denmark; 9 Department of Agronomy, Federal University of Viçosa, Viçosa, Minas Gerais Brazil; 10 International Potato Center (CIP), Lima 12, Apartado, 1558 Peru; 11 Department of Horticulture Science, NC State University, Raleigh, NC 27695 USA; 12 USDA ARS Horticultural Crops Production and Genetic Improvement Research Unit, Corvallis, OR 97333 USA; 13 Savanna Institute, 2453 Atwood Ave. Suite 209, Madison, WI 53704 USA; 14 USDA ARS Plant Germplasm Introduction and Testing Research Unit, Prosser, WA 99164 USA; 15 Value Crop Research Institute, National Institute of Crop and Food Science, Rural Development Administration, Muan, 58545 Republic of Korea; 16 Chiayi Agricultural Experiment Branch, Taiwan Agricultural Research Institute, Ministry of Agriculture, Chiayi, Taiwan; 17 Department of Agronomy and Plant Genetics, University of Minnesota, Saint Paul, MN 55108 USA; 18 USDA ARS Genetic Improvement for Fruits & Vegetables Laboratory, Chatsworth, NJ 08019 USA; 19 USDA ARS Dairy Forage Research Center, Madison, WI 53706 USA; 20 USDA ARS, Plant Science Research Unit, St. Paul, MN 55108 USA; 21 USDA ARS Agricultural Water Efficiency and Salinity Research Unit, Riverside, CA 92507 USA; 22 International Potato Center (CIP), Ntinda II Road, Plot 47, Kampala, Uganda; 23 USDA ARS US Vegetable Laboratory, Charleston, SC 24914 USA; 24 College of Agriculture, Science and Education, Faculty of Food and Agriculture Department of Plant, Soil Sciences, and Engineering, Passley Gardens, P.O. Box 170, Port Antonio, Portland, Jamaica; 25 Pathology, and Genetics Research Unit, USDA ARS Soybean/Maize Germplasm, Urbana, IL 61801 USA

## Abstract

**Keymessage:**

Standardized microhaplotype databases for eight diverse crops enable multiallelic analyses, comparative genetics, and breeding decisions.

**Abstract:**

Microhaplotypes are short genomic segments that contain multiple tightly linked variants, providing multi-allelic data that can enhance genetic resolution compared to traditional biallelic single nucleotide polymorphism (SNP) markers. Here, we present the creation and utilization of separate microhaplotype databases for eight crop species representing diverse genome sizes, ploidy levels, and breeding systems. We developed a standardized, species-agnostic pipeline for processing, filtering, and databasing microhaplotypes generated using the DArTag targeted genotyping platform. To enhance user accessibility, we developed a no-code, user-friendly application, HapApp, that uses an R Shiny front-end interface to allow breeders and researchers to add unique, standardized microhaplotype identities from raw DArTag reports and iteratively update the existing crop-specific database with the newly discovered microhaplotypes. Selected case studies with these databases highlight the operational advantages of microhaplotypes, especially for challenging, highly heterozygous, or polyploid species. They offer an informative alternative to traditional biallelic SNP analyses for resolving population structures and improving linkage map ordering. This integrated framework provides a reproducible and scalable foundation for managing and exploiting microhaplotype data in plant breeding and genetic research, enabling robust cross-project comparisons and facilitating trait discovery in both simple and complex crop genomes, while enabling comparative genomics and cross-species functional transfer that accelerates genetic gains across all crop species.

**Supplementary Information:**

The online version contains supplementary material available at https://doi.org/10.1007/s00122-026-05340-4.

## Introduction

The integration of molecular markers into plant breeding programs has dramatically accelerated genetic improvement by enabling precise germplasm characterization and selection. DNA-based markers in plants have taken many forms over the past several decades, such as restriction fragment length polymorphisms (RFLPs), random amplified polymorphic DNA (RAPD), amplified fragment length polymorphisms (AFLPs), simple-sequence repeats (SSRs) (Mrázek et al. 2007), and single nucleotide polymorphisms (SNPs) (Botstein et al. 1980; Williams et al. 1990; Vos et al. 1995; Mrázek et al. 2007; Mammadov et al. 2012). Today, the predominant DNA marker type is the biallelic SNP marker due to its abundance and wide distribution across genomes and its suitability for high-throughput genotyping. SNPs can be identified cheaply and quickly using a wide range of detection technologies. For researchers, SNP datasets require minimal storage space and are easier to interpret than many other marker systems. There are several software packages available for researchers to use that aid allele calling from raw data, including proprietary software such as KlusterCaller and SNPviewer (LGC Ltd., Huntingdon, UK) and GenomeStudio (Illumina, San Diego, CA, USA), and free R packages such as polyRAD and updog (Clark et al. 2019; Gerard and Ferrão 2020). SNPs are often preferred for time-sensitive, data-driven decisions on germplasm in breeding programs. As such, SNPs have become indispensable for assessing genetic diversity and population structure, mapping traits of interest, and implementing marker-assisted selection/genomic selection in plant and animal breeding programs. While biallelic SNP calling is often sufficient and accurate for diploids or species with low levels of heterozygosity, it lacks sufficient information to accurately resolve genotypes in highly heterozygous species and species with complex genomes, especially polyploids. Recent advances in DNA sequencing technology have led to the development of various approaches for genetic architecture profiling. Whole-genome sequencing (WGS) provides the most comprehensive view of genetic variation but remains cost-prohibitive to be applied yearly for routine breeding applications. To address this limitation, reduced-representation approaches have emerged as cost-effective alternatives. These methods can be broadly categorized into two strategies: random sampling and targeted amplification of genome sequences.

Random reduced-representation methods, such as genotyping-by-sequencing (GBS) and Diversity Arrays Technology sequencing (DArTseq), employ restriction enzymes to sample a subset of the genome (Elshire et al. 2011; Cruz et al. 2013). GBS typically utilizes methylation-sensitive restriction enzymes to reduce genome complexity, thereby enriching for low-copy and genic regions and increasing the likelihood of detecting functional variants. In DArTseq, a species-specific combination of restriction enzymes is used to achieve genome complexity reduction and preferentially amplify low-copy fragments. Both approaches generate reduced-representation libraries and have proven particularly valuable for species with limited or no reference genome resources, enabling rapid and cost-effective genome-wide variant discovery. The downside of both methods is the low reproducibility from one project to another, since a random subset of a reduced-representation library is sequenced each time, leading to high missing data rates between project runs. Additionally, the data analysis is computationally intensive, requiring skilled bioinformatic support and ample time to complete.

In contrast, targeted genotyping approaches such as DArTag, AgriSeq, FlexSeq, GT-seq, and related platforms are typically designed with primers flanking an anchor SNP at each locus (Campbell et al. 2015; Semalaiyappan et al. 2022; Blyton et al. 2023; Clare et al. 2024). These methods offer several advantages over GBS and DArTseq, including higher coverage of target loci, low missing data rates, read depths that are consistent across samples and projects ensure data are comparable across projects, and reduced computational requirements for data analysis (Zhao et al. 2023, 2024b, a; Hardigan et al. 2023; Sandercock et al. 2025). A further key advantage is the ability to generate detailed sequence information surrounding variant sites, as these approaches employ next-generation sequencing of short reads (typically 50–250 bp). Rather than providing only single nucleotide calls at target variant sites, these platforms return data in two formats: raw FASTQ files containing individual sequencing reads and semi-processed marker reports presenting multi-allelic sequences for each locus. These sequences contain not only the target variant site but also capture adjacent polymorphisms within the amplified regions. The co-occurrence of closely linked variants within these short haplotype blocks can yield three or more allelic combinations, characterizing a multi-allelic microhaplotype (Kidd et al. 2013). Due to their short length, variants within a microhaplotype are assumed to remain in strong linkage and be inherited as a unit, and the combination of variants within have known phasing. Multi-allelic genetic data can provide more detailed and nuanced insights into genetic diversity, allele dosage, trait expression, and population structure. The comprehensive sequence information also enables more accurate genotype calling through sequence-context evaluation, enhanced discrimination power through microhaplotype-based analysis, and the identification of novel alleles within the amplified regions.

The concept of microhaplotypes was first introduced in the forensics field by Kidd and colleagues (Kidd et al. 2013) and rapidly gained popularity due to their high information content and analytical efficiency. Subsequent studies in forensic genetics have demonstrated the advantages of using multi-allelic microhaplotypes over individual SNPs in diverse applications, including kinship investigations, ancestry inference, resolution of complex DNA mixture, and recovery of allelic information from degraded DNA specimens (Kidd et al. 2014; Oldoni and Podini 2019; de Barros Rodrigues et al. 2025; Turchi et al. 2026). Beyond forensics, the use of microhaplotypes derived from targeted genotyping panels has demonstrated notable advantages in aquaculture. GT-seq microhaplotypes have been successfully applied to improve parentage assignment in oysters, salmon, and rockfishes (Baetscher et al. 2018; Thompson et al. 2025; Anderson et al. 2025). For example, simulation studies in oyster and salmon breeding programs have indicated that incorporating microhaplotypes can increase the accuracy of estimated breeding values (Delomas et al. 2024).

In plant breeding, the advantages of using multi-allelic markers have been highlighted across diverse applications, including genomic prediction (GP) and genomic selection (GS) as well as genome-wide association studies (GWAS) (Matias et al. 2017; Abed and Belzile 2019). Traditionally, most multi-allelic markers in crop studies have been derived from GBS or WGS datasets by estimating combinations of SNPs in complete linkage disequilibrium, referred to as haplotype blocks. Such blocks are typically inferred through methods such as confidence interval-based approaches using normalized measures of allelic association, identification of genomic regions with limited recombination events, or read-based phasing approaches (Gabriel et al. 2002; Wang et al. 2002; Garrison and Marth 2012; Martin et al. 2016). Simulation studies have shown that GWAS models accommodating multi-allelic markers and haplotype blocks in polyploid species achieve higher accuracy than models based on biallelic SNPs (Thérèse Navarro et al. 2022). More recently, microhaplotypes derived from DArTag panels have been applied to genomic studies in alfalfa, demonstrating higher values of both intra- and inter-population diversity compared to SNP markers (Medina et al. 2025).

Over the past five years, Breeding Insight (BI; RRID: SCR_026645) and collaborators have developed a series of medium-density DArTag genotyping panels (Zhao et al. 2023, 2024b, a, 2025; Hardigan et al. 2023; Endelman et al. 2024; Nayak et al. 2025; Chen et al. 2025, 2026). These panels were developed using a diverse breeding germplasm and validated across F_1_ and/or backcross mapping populations as well as core diversity panels, ensuring robustness across breeding materials. The DArTag genotyping platform produces several data outputs including traditional FASTQ files and a proprietary format called Missing Allele Discovery Counts (MADC) file. The MADC file contains read counts for all microhaplotypes at targeted loci and captures four distinct classes of microhaplotypes. Reference (Ref) and Alternative (Alt) alleles represent the target variants known a priori from panel design and are analogous to SNP marker definitions of the same designation. In contrast to Ref and Alt, RefMatch and AltMatch sequences match either the reference or alternative state at the target site while containing additional polymorphisms in the flanking sequences that were not known at the time of design, thus newly ‘discovered.’

One challenge in utilizing MADC data across projects stems from the lack of unique identifiers for RefMatch and AltMatch alleles from all genotyping projects. To systematically organize the RefMatch and AltMatch information, we have established a species-agnostic pipeline to assign standardized and unique microhaplotype identities (IDs) to serve three essential purposes: 1) enable efficient tracking of marker genetic information, 2) facilitate cross-project and cross-institution comparison of genotyping results, and 3) abide by Findable, Accessible, Interoperable, and Reusable (FAIR) data principles (https://www.go-fair.org/fair-principles/).

Crucially, this framework has allowed us to compile and curate a massive foundational set of data assets. Here, we present the individual microhaplotype databases for eight plant species (alfalfa, blueberry, cranberry, cucumber, pecan, potato, strawberry, and sweetpotato). These public databases serve as the primary resource enabling breeders and researchers to systematically assess, compare, and mine global crop genetic diversity through unified, multi-allelic community efforts.

## Materials and methods

### Datasets used to generate the microhaplotype databases

The eight microhaplotype databases described in this work were populated by compiling mid-density targeted genotyping data from an extensive, collaborative network of domestic and international programs, including researchers from the United States Department of Agriculture, Agriculture Research Service (USDA-ARS), multiple public land-grant universities, international agricultural centers, and private commercial breeding firms. To secure broad community participation and comply with disparate and strict institutional germplasm protections, all data-sharing agreements were operated under a confidentiality clause: Individual sample identifiers, raw passport descriptors, and specific pedigree information were blinded prior to populating the databases. While granular sample-level metadata cannot administratively be disclosed, the databases capture the robust aggregate allelic diversity generated across these extensive genetic mapping and diversity channels.

Diverse sample sets from each species were collected by these contributing breeders and researchers, genotyped using the DArTag platform independently at DArT (Diversity Arrays Technology, Bruce, Australia; www.diversityarrays.com), and the resulting MADC reports were shared by collaborators with BI to generate the microhaplotype databases. To provide the necessary genetic context for evaluating database structure, the biological materials are classified by aggregate metrics that describe breeding-use categories across each crop species.

The alfalfa (*Medicago sativa* L.) microhaplotype database was created using genotyping data from 25 distinct USDA-ARS and university breeding projects, totaling over 15,600 samples. The material spans diverse diploid annual *Medicago* species from the National Plant Germplasm Repository (Zhao et al. 2024c), autotetraploid structured polycross populations (targeting traits like stem digestibility, winter survival, drought tolerance, spreading, yield), synthetic varieties, genomic selection populations, and core breeding lines representing the US alfalfa breeding community.

The blueberry (*Vaccinium corymbosum* and related *Vaccinium* subgenus species from the *Cyanococcus* section) microhaplotype database was generated by compiling genotyping reports of ~ 8,930 samples, which consisted of wild *Vaccinium* germplasm from the section of *Cyanococcus*, hexaploid rabbiteye, tetraploid highbush blueberry material, predominantly *V. corymbosum* breeding lines, F_1_ mapping populations, and advanced selection lines containing minor introgressions from wild relatives.

The cranberry (*Vaccinium macrocarpon* Aiton and related *Oxycoccus* subgenus species from the *Oxycoccus* section) microhaplotype database was built from over 4 K samples from mostly diploid cranberry. This includes diversity, F_1_ and backcross mapping populations alongside specific breeding selections. Notably, a tetraploid blueberry × cranberry hybrid population was also included.

The cucumber (*Cucumis sativus* L.) microhaplotype was derived from over 8 K samples of diploid material. The source material was obtained from multiple biparental F_1_, backcross populations, key breeding parents, and near-isogenic lines.

The pecan (*Carya illinoinensis*) microhaplotype database was generated from more than 8 K samples of diploid material. The source material was obtained from multiple biparental F_1_ and backcross populations tied directly to active breeding programs, as well as key breeding founders and other *Carya* species.

The sweetpotato (*Ipomoea batatas* L.) microhaplotype database represented a massive global footprint across 7 independent international projects encompassing more than 9 K samples. This cohort evaluated hexaploid breeding germplasm distributed across North America (US), Africa (Mozambique, Nigeria, Uganda), Asia (South Korea and Taiwan), South America (Peru), and Central America/the Caribbean (Costa Rica and Jamaica). The materials include structured breeding populations, key breeding parent lines, and diverse accessions that are country or market specific.

The potato (*Solanum tuberosum* L.) microhaplotype database was derived from over 3 K samples sourced from USDA-ARS and university programs. This included samples from cultivated potato, key parental lines, hybrid populations between *S. tuberosum* and *S. microdontum*, wild *Solanum* species, and cultivated potato relatives.

The strawberry (*Fragaria* × *ananassa*) microhaplotype database was created from nearly 2 K samples from the USDA-ARS National Clonal Germplasm Repository (Corvallis, OR). This dataset contained samples from various species, subspecies, forms, and hybrids within the genus *Fragaria*, with the most abundant samples from *Fragaria* × *ananassa* and *Fragaria chiloensis*.

### Core database structure and panel-specific considerations

The core database forms the foundation of the microhaplotype tracking system, offering consistent reference points for allele comparison and standard templates for sequence alignment. This core database contains both Ref and Alt alleles, with sequence lengths varying according to panel design specifications. The genomic insert size in DArTag panels has lengthened over time as sequencing costs have decreased, resulting in two main design categories. The 54-bp design (~ 75 bp with primer sequences before trimming) was implemented in alfalfa, blueberry, and Phase I markers in potato and strawberry panels. The 81-bp design (~ 100 bp with primer sequences before trimming) was implemented in cranberry, cucumber, pecan, sweetpotato panels, and Phase II markers in potato and strawberry panels.

### Construction of reference-based core database

The construction of the core microhaplotype database for reference-based markers involves an iterative process using probe design files, reference genomes, and analytical scripts. This workflow addresses two key challenges: 1) In the genotyping reports, Ref and Alt amplicons often contain International Union of Pure and Applied Chemistry (IUPAC) codes representing ambiguous base calls, and 2) the 3’ ends of some amplicons may contain sequencing errors. The primary goal is to create a core database of Ref and Alt microhaplotypes that are free of IUPAC codes and mismatches (except for the target variant in Alt) with the reference genome. Another critical consideration is that a DArTag panel, like other amplicon-sequencing technologies, is composed of amplicons derived from both the top and bottom strands of the reference genome. Accurately establishing the strand orientation is crucial for determining the precise positions and nucleotides of the variant sites. To maintain consistency, all microhaplotype databases preserve strand orientation as implemented during panel creation by DArT.

The workflow begins by extracting 180–300 bp flanking sequences encompassing the target variants from the corresponding reference genome (Table 1). Ref and Alt amplicon sequences are then retrieved from the DArTag MADC report and aligned to the flanking sequences via BLAST (Camacho et al. 2009). This alignment step helps determine the correct strand orientation of the amplicons, and those amplicons originating from the bottom strand are exported as a key file for downstream analysis (Table S1). Meanwhile, the coordinates of the Ref and Alt microhaplotypes are determined based on the reference genome, and sequences are fetched to populate the core database. By design, the core database contains twice the number of marker loci in a DArTag panel, as it incorporates both Ref and Alt microhaplotype sequences for each locus.

**Table 1:** Summary of DArTag panels and microhaplotype databases across eight species

Species	Ploidy	Reference genome	Developed by	Design length	#Loci	Database version^c^	Microhaplotypes	Samples
Alfalfa	4x	(Chen et al. 2020)	BI^b^	54 bp	3000	50	35,259	15,600
Blueberry	4x	(Colle et al. 2019)	BI	54 bp	3000	20	28,653	8930
Cranberry	2x	(Diaz-Garcia et al. 2021)	BI	81 bp	3050	5	14,380	4146
Cucumber	2x	(Yang et al. 2012)	BI	81 bp	3059	10	9823	8272
Pecan	2x	(Lovell et al. 2021)	BI	81 bp	3100	9	26,073	6768
Potato	4x	(Pham et al. 2020)	J. Endelman	54–81 bp	3913	15	35,741	3102
Strawberry^a^	8x	(Hardigan et al. 2021)	M. Hardigan/S. Knapp	54–81 bp	5000	3	38,227	1880
Sweetpotato	6x	(Wu et al. 2018)	BI	81 bp	3120	19	37,593	9212

^a^Panel designed for cultivated strawberry (octoploid, 8x) where markers were sub-genome specific, enabling functional diploid analysis

^b^BI: Breeding Insight

^c^Database version numbers increase when a new genotyping project is processed and/or after removing duplicated entries in the database

### Reference genome mapping and standardized marker IDs

To ensure naming consistency across DArTag panels developed at different times and by independent research groups, we implemented a systematic marker identity (ID) standardization process prior to microhaplotype ID assignment. The original marker IDs were converted to a uniform format that begins with the chromosome number (or scaffold designation), and underscore separator for improved parsing, followed by the exact genomic position derived from the reference genome utilized for the initial panel design. All genomic positions were left-padded to nine digits to maintain consistent alphanumeric formatting (e.g., Chr01_000589632).

All reference genomes chosen for the eight crop species were highly contiguous, chromosome-scale assemblies (Table 1). In polyploid genomes (e.g., alfalfa, blueberry, and strawberry), haplotype-phased reference assemblies were preferentially utilized to resolve sub-genomic locations if available such genomes were publicly available at the panel design stage. Specifically, strawberry markers are mapped to the four sub-genomes (A, B, C, and D) of the octoploid reference genome to maintain sub-genome specificity following the naming conventions of each crop. For example, in strawberry we converted the original panel marker ID ‘FanaDarT_P2_M00419’ to the standardized ‘chr_1B_019995133.’ For tetraploid alfalfa (e.g., chr1.1_000194324) and blueberry (e.g., Chr07_000013166), all the markers are derived exclusively from the first sub-genome of the reference assemblies in Table 1. For all other species panels, a single haploid reference assembly was used for marker mapping as shown in Table 1. For markers situated on unanchored scaffolds or non-reference sequences, the uniform naming structure substitutes the chromosome string with the corresponding scaffold or sequence ID identifier (e.g., Rychc_M6v5_chr09_037964457).

### Database versioning and backward compatibility

Incremental database updates accommodate newly discovered microhaplotypes without altering these foundational marker IDs. Database versions increase dynamically when processing a new genotyping project that yields novel microhaplotypes meeting our strict inclusion thresholds or following systematic removal and merging of duplicated sequence entries during processing.

To ensure long-term database stability, these coordinate-based marker IDs remain fixed to the design assembly version. In the event of future community-wide reference genome updates, portability and backward compatibility are preserved via the invariant 180–300 bp flanking sequences encompassing each target variant. These sequence anchors allow legacy marker IDs to be systematically cross-mapped to new assembly coordinates via local sequence alignment (BLAST) (Camacho et al. 2009).

### Quality control and database updates

**Fig. 1:**
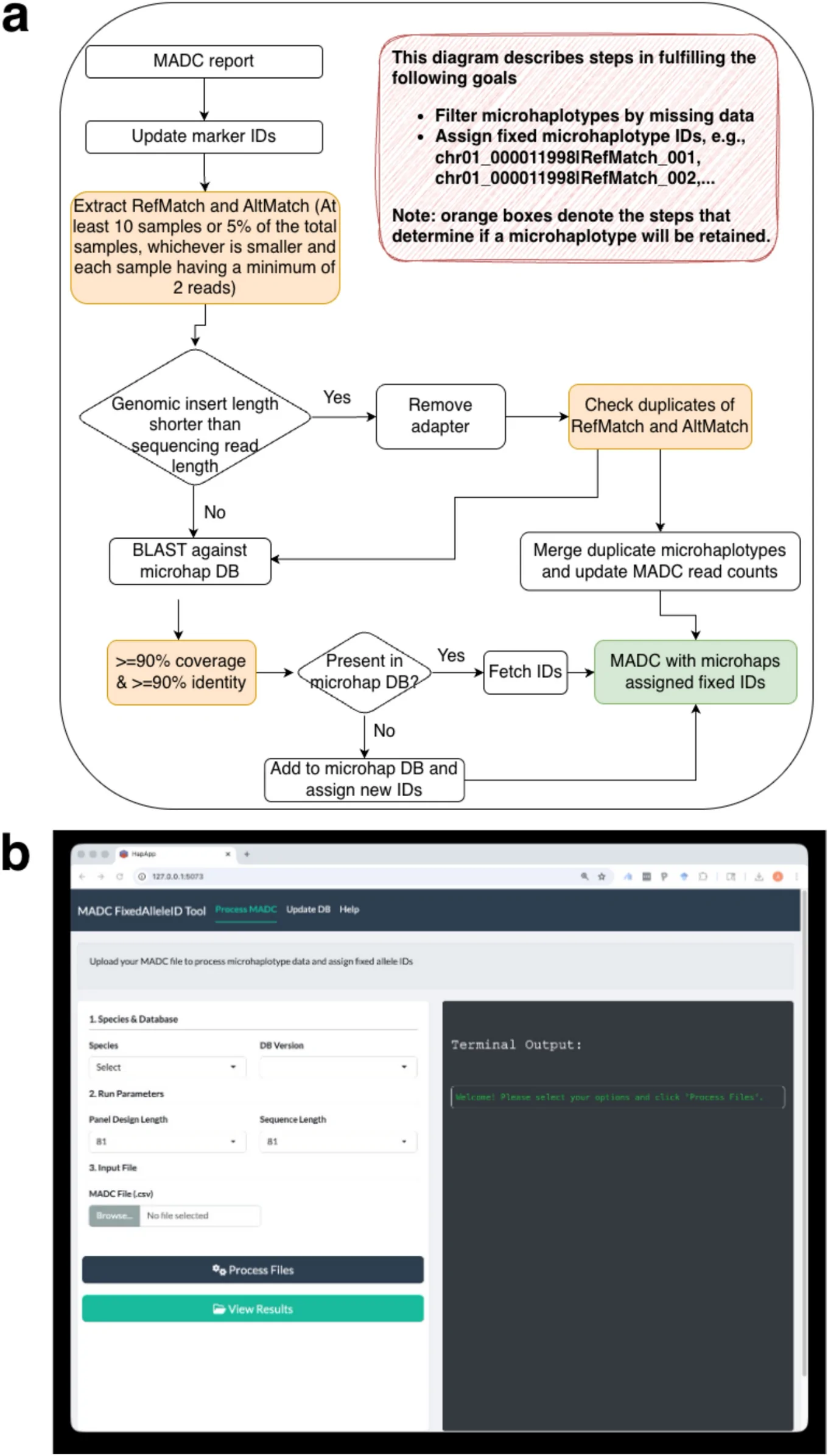
HapApp workflow and interface for fixed microhaplotype IDs. **a** Schematic of the workflow to filter microhaplotypes, assign unique fixed microhaplotype IDs, and update the microhaplotype database using MADC genotyping reports. **b** Screenshot of the HapApp R Shiny interface for processing and ID assignment

The workflow begins by updating the initial MADC report into this standard chromosome_000000000 format. As an initial quality control, we filter RefMatch and AltMatch sequences to require a minimum presence of either 10 samples or 5% of the total samples in a project (whichever is smaller), with each sample having at least two reads (Fig. 1a).

The workflow diverges from this point based on amplicon size. For reports containing sequencing reads longer than genomic insert size by panel design, the reads often contain adapter sequences. In this case, microhaplotypes were first cleaned by removing potential adapter sequences with Cutadapt (Martin 2011), and any duplicates identified among RefMatch and AltMatch sequences are marked for exclusion. For reports with reads matching the panel design length, no adapter removal is needed. All sequences underwent BLAST analysis against the existing microhaplotype database to determine exact sequence matching and database inclusion (Camacho et al. 2009). Crucially, an amplicon sequence was only mapping to a previously cataloged database ID if it shared 100% identity and coverage with an existing entry, producing an updated MADC report with fixed microhaplotype IDs. Any sequence that did not achieve a single 100% match but satisfied our minimum quality filter (≥ 90% identity over ≥ 90% coverage of subject microhaplotypes) was recognized as a newly discovered variant, incorporated into the database, and assigned a unique ID following the RefMatch and AltMatch classification scheme. Any sequence failing to meet the above minimum filter was discarded as a technical artifact or off-target amplification. These new unique IDs were then written back to the updated MADC report, which then had unique standardized IDs for all observed microhaplotypes. The process yielded two key outputs: (1) MADC report with standardized universal microhaplotypes IDs (MADC_fixedID) and filtered for missing data and (2) a new version of the microhaplotype database incorporating newly identified alleles with unique IDs. If no new alleles were found, the database remained unchanged, but the MADC report is still generated with universal standardized microhaplotype IDs.

It is important to note that the initial filtering threshold (≥ 90% identity) establishes a generous sequence alignment window that can accommodate ~ 5 mismatches in 54 bp designs and 8 mismatches in 81 bp designs. While this broad threshold ensures that novel variants from diverse sources (e.g., highly polymorphic wild relatives) are captured, it presents a risk of inappropriately grouping conserved paralogous sequences. Rather than filtering these sequences out, it is best to capture and systematically annotate them as putatively paralogous (see below for the test cases in alfalfa and blueberry) because they are likely to recur in subsequent genotyping projects. Depending on the specific downstream application, automated data-driven biological and software filters can be deployed to exclude these multi-locus artifacts. For example, in a classic biparental population, the maximum possible allele count is biologically capped at four for loci where both parents are heterozygous. Furthermore, genotype-calling and linkage mapping software contain internal segregation and data quality metrics that automatically flag and filter out poorly behaving markers, which include multi-locus amplification. Together, these safeguards ensure that while the global database maintains a comprehensive catalog of all sequence variants to accommodate polymorphic sources like wild relatives, researchers can isolate true orthologous allelic counts to minimize technical noise for downstream analyses.

### Creation of an R Shiny interface for processing standardized microhaplotype ID assignment

The workflow described above provides a flexible pipeline for processing MADC files, identifying new unique microhaplotypes, and producing an updated MADC with fixed microhaplotype IDs based on the microhaplotype database. However, its Bash and Python scripting format makes it inaccessible to users without coding experience or who are uncomfortable with their skill level. To improve user accessibility, we created a no-code R Shiny interface that provides a point-and-click method for using the workflow (Chang et al. 2024), which has been shown to provide complex plant and animal breeding workflows with a more approachable framework (Fig. 1b; Sandercock et al. 2025). When the original MADC file is uploaded, it is first checked to confirm it is in the correct format. Then, the application submits the MADC to the pipeline described above, which performs the computation steps. The updated information is captured and displayed to the user along with any warnings or notifications. The output files include 1) a new version of the microhaplotype FASTA file and 2) a MADC file with microhaplotypes that passed initial quality control and were assigned a unique standardized ID.

### Sequence alignment for the alfalfa and blueberry microhaplotype databases

The alfalfa microhaplotype database, as of November 2025, is at v50. It was aligned to the haplotype-based ‘XinJiangDaYe’ reference genome (Chen et al. 2020) to understand the distribution of potential paralogous amplifications. The alignment was performed using Bowtie2 v2.5.1 (Langmead and Salzberg 2012) with default parameters. All possible alignments were returned followed by a preliminary filter to remove any alignment with a total number of insertions/deletions > 5 bp. A total of 34,891 microhaplotypes were successfully aligned to at least one locus in the reference genome. The syntenic genomic regions (Chen et al. 2020) across four haplotypes in each of the eight homologous groups were used to determine the most likely syntenic alignment when a microhaplotype aligned to multiple loci on a single haplotype. A microhaplotype was considered a potential paralogous amplification if it had 1) one or more alignments outside its target homologous group or 2) two or more copies on any of the four haplotypes within the homologous group but outside the target genomic region (Fig. S1c & d). For DArTag loci containing only non-paralogous microhaplotypes, the copy number (ranging from one to four) of each target locus was determined based on the number of aligned positions within the corresponding syntenic regions (Fig. S1 a & b).

The most up-to-date blueberry microhaplotype database, v20, was characterized for potential paralogous amplification using the same strategy as in alfalfa. The blueberry database was aligned to ‘Draper’ reference genome (Colle et al. 2019), and 28,448 microhaplotypes were successfully aligned to at least one locus in the reference genome. Potential paralogous amplifications and copy number of non-paralogous loci in the blueberry microhaplotype database were determined as described above.

### Comparison of microhaplotypes, target, and off-target markers through linkage map construction in pecan

Linkage maps were built using OneMap v3.2.2 (Taniguti et al. 2022) based on a previously published pecan (*Carya illinoinensis*) F_1_ dataset comprising 188 progeny (Chen et al. 2026). The mapping procedure was applied using three different data set marker types: (i) microhaplotypes, (ii) target SNPs only (target-SNPs-dataset), and (iii) combined target and off-target SNPs (all-SNPs-dataset).

For the microhaplotype dataset (i), the MADC file was processed using our standard workflow, which assigns fixed microhaplotype IDs based on the pecan microhaplotype database v9. The processed file was converted to a polyRAD (v2.0.0) input object using the *readDArTag* function, and genotypes were called using the *IterateHWE* function, which assumes Hardy–Weinberg equilibrium without population structure (Clark et al. 2019). We used this approach instead of *PipelineMapping2Parents* to avoid errors that can arise when parental genotypes are altered or imputed from progeny segregation patterns (Taniguti et al. 2022). The resulting genotypes were exported as a Variant Call Format (VCF) file using the *RADdata2VCF* function with asSNP parameter set to FALSE. For datasets (ii) and (iii), VCF files were generated from the MADC file with the *madc2vcf_targets* and *madc2vcf_all* functions from the BIGr (v0.6.2) package (Sandercock et al. 2025), respectively. These VCFs contained allele count data and were processed in polyRAD under the same Hardy–Weinberg equilibrium (HWE) genotype-calling model used for dataset (i). All datasets were subjected to the same filtering criteria: a minimum genotype depth of six reads, a mean marker depth greater than 30, a maximum of 25% missing genotypes per marker and per individual, removal of redundant markers that presented identical genotype patterns across all individuals (treated as belonging to the same LD block), and exclusion of markers deviating from expected Mendelian segregation (*α* = 0.05, Bonferroni-corrected for multiple tests).

Additional filtering was performed using the *rf_snp_filter_onemap* function, which assesses pairwise recombination fractions (rf) and LOD scores to identify informative markers. For each pair of markers, the function retained only comparisons with strong linkage support (LOD > 5) and low recombination (rf < 0.15). Pairs that did not meet these criteria were temporarily masked (set to missing), ensuring that only informative pairwise combinations contribute to the count. The number of remaining informative (non-missing) pairwise comparisons were then counted for each marker, and markers falling below the 5th percentile or above the 95th percentile of the resulted counts distribution were removed.

Markers were assigned to linkage groups according to their chromosome in the reference genome. Linkage distances were estimated considering the order of markers using either their physical positions or the multidimensional scaling (MDS) ordering algorithm (Preedy and Hackett 2016). When MDS ordering failed due to collinearity, the lower quantile threshold in *rf_snp_filter_onemap* was relaxed. In dataset (ii), the threshold was raised to 0.40 for linkage group (LG) 2 and to 0.35 for LG 10 and LG 11. In dataset (iii), the threshold was raised to 0.35 for LG 2 and LG 11. The performance of MDS ordering algorithm in each dataset was evaluated by computing the absolute value of Spearman’s rank correlation coefficient ρ between the algorithm-derived marker order and the reference genome order.

The correlation coefficient was calculated according to Spearman (1904) using the formula

$$\rho = 1 - \backslash frac\left\{ {6\mathop \sum \limits_{{\left\{ {i = 1} \right\}}}^{{\left\{ m \right\}d_{i}^{2} }} } \right\}\left\{ {m\left( {m^{2} - 1} \right)} \right\},$$

where *d*_*i*_ denotes the rank difference for marker *mi* between the estimated and true orders.

### Comparative analysis of microhaplotype and SNP genotyping data in sweetpotato breeding germplasm

Microhaplotype variant data were generated from MADC reports in which alleles had been assigned unique standardized IDs across seven independent sweetpotato DArTag genotyping projects. For each MADC dataset, only Ref and Alt microhaplotypes were retained for marker loci with greater than 15 microhaplotypes since they likely contained some paralogous amplification. Microhaplotype genotype dosage calls were produced as VCF using the *readDArTag* function in the polyRAD (v2.0.0) package (Clark et al. 2019). Individual project VCF files were merged to form a single consolidated VCF and subjected to sequential quality filtering. Variants with low coverage (mean read depth < 10), excessively high coverage (mean read depth > 1000), or failing missingness criteria were filtered using a population-aware strategy. A microhaplotype was retained if it met either the global threshold of ≤ 20% missing data across all samples or the per-population threshold of ≤ 20% missing data in at least one population. This approach ensures that markers informative for specific populations, including low-frequency or private alleles, are not discarded due to varying population size or poor performance in other populations. After dosage calling and marker filtering, samples with ≤ 20% missing data were removed. Given the multi-allelic nature of microhaplotype genotypes, conventional minor allele frequency (MAF) was not an appropriate measure. Instead, a combined alternative allele frequency (AAF) was calculated and used to filter loci, with those having AAF below 0.05 excluded from further analysis. All filtering steps were performed under a hexaploid ploidy assumption, consistent with the known sweetpotato genome structure. The resulting microhaplotype dataset represented the union of high-quality loci across all projects and was used for principal component analysis (PCA) and discriminant analysis of principal components (DAPC) to evaluate population structure and assignment accuracy using the R packages vcfR (v1.15.0) and adegenet (v2.1.11) (Jombart 2008; Knaus and Grünwald 2017).

Two SNP datasets were generated, target-SNPs-dataset and all-SNPs-dataset (target + off-target SNPs). For SNP analysis, loci absent from the final filtered microhaplotype dataset were first removed from all per-project MADC files to ensure that downstream SNP calls were drawn from the same set of genomic regions. Individual project SNP genotypes were generated using the *readDArTag* function with the parameter asSNPs set to TRUE in polyRAD (v2.0.0) package. The resulting SNP datasets were merged into a single SNP VCF under the same hexaploid model. Samples not present in the final filtered microhaplotype dataset were excluded from the concatenated SNP dataset, resulting in identical sample representation across data types, regardless of marker type (e.g., microhaplotype or SNP). Except removal of monomorphic sites, no additional filters were applied on the all-SNPs-dataset to maintain comparability to microhaplotypes. Target-SNPs-dataset was obtained from the all-SNPs-dataset and underwent the same filters as microhaplotypes. All three marker sets, including microhaplotypes, all-SNPs-dataset, and target-SNPs-dataset, comprised of 4,087 matched samples, which were then processed through equivalent PCA and DAPC pipelines.

## Results

### Creation of an R Shiny interface for standardizing unique microhaplotype ID assignment

We developed an R Shiny application, HapApp (v1.0), that provides a no-code option for users to process their MADC files, assign fixed microhaplotype IDs, and output an updated version of the microhaplotype FASTA file. The interface allows the user to customize the pipeline depending on their genotyping panel attributes such as species, microhaplotype length, and panel design (Fig. 1b). The most recent microhaplotype databases for each of the above-mentioned species (alfalfa, blueberry, cucumber, cranberry, potato pecan, strawberry, and sweetpotato) are available with the app, and updated versions retrievable by the user through the ‘Get Database’ function in HapApp. Since the app directly uses the Bash/Python pipeline described above, our testing showed HapApp to be reliable for processing of MADC files in a no-code framework.

### Descriptive scale and architecture of the foundational databases

We compiled and curated eight crop microhaplotype databases that vary across genome complexities, ploidy levels, and reproductive systems (Table 1). These public data assets represent an extensive curation effort capturing tens of thousands of unique microhaplotypes across more than 60,000 community samples globally. Summary statistics reveal that while the databases collectively showcase rich allelic diversity, the raw allele metrics and database growth are strongly shaped by technical panel attributes and sampling scales alongside baseline biology.

**Fig. 2:**
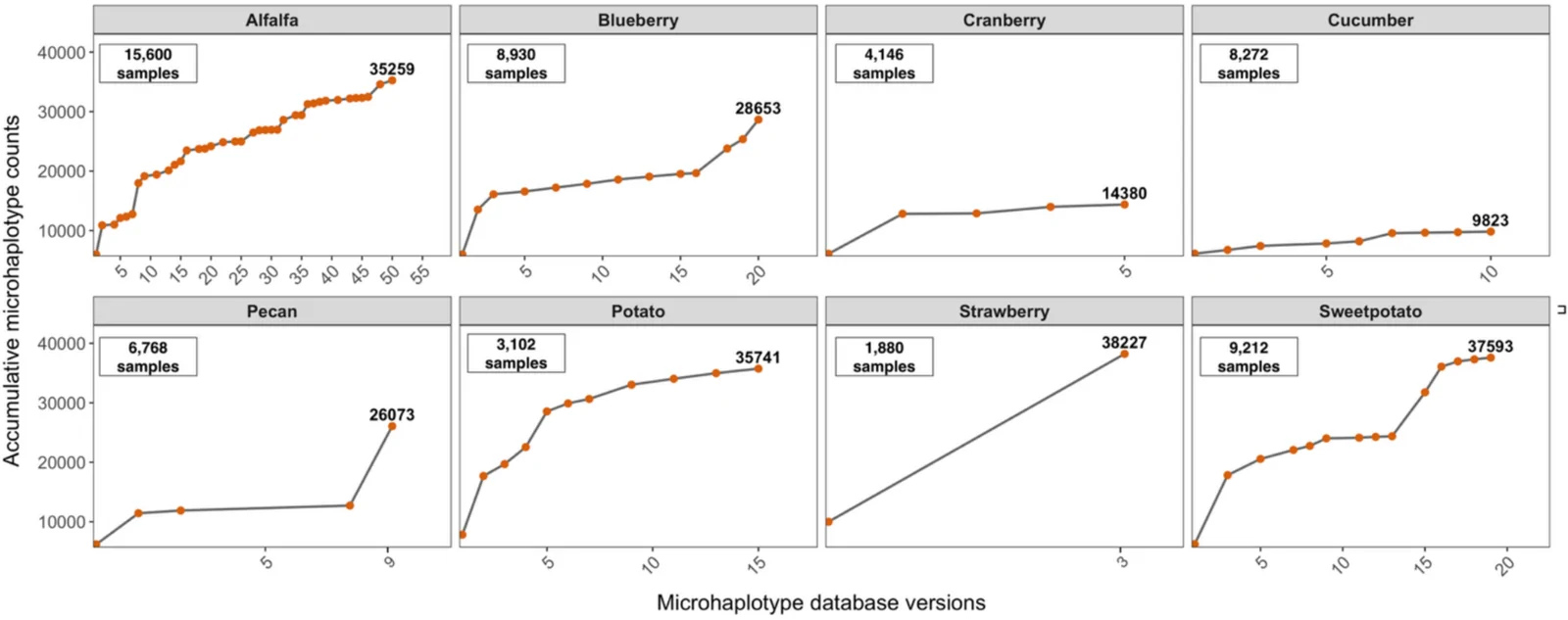
Accumulative allele counts per microhaplotype database across eight crop species

For the DArTag panels of all eight species, marker loci were broadly distributed across all chromosomes with greater density in genic regions. As described in the Materials and Methods, eight microhaplotype databases were built following the standardized approach for unique ID convention, filtering, and appending new alleles to databases. Each database consists of RefMatch and AltMatch microhaplotypes in addition to Ref and Alt microhaplotypes (54–81 bp) by panel design. It should be noted that not all microhaplotypes are orthologous to the assay design; some paralogous microhaplotypes may also be included, especially in species with high genome duplication rates or autopolyploidy. Here, we will first provide a brief description of microhaplotype databases for four crops (cranberry, cucumber, potato, and strawberry) and highlight the annotation, usage, and advantages of using microhaplotypes over SNPs for four other crops (alfalfa, blueberry, pecan, and sweetpotato) (Fig. 2).

### Cranberry microhaplotype database

The cranberry microhaplotype database (v5) consists of 14,380 alleles from 3050 loci (Fig. 2). These were genotyped from 4146 samples. This represents the latest and most comprehensive version (cranberry 3 K DArTag v2.0) of the database, with loci evenly spread across the 12 chromosomes. This version was built on improvements made to the initial v1 panel (Chen et al. 2025), addressing important issues of marker proximity and integrating new markers that cover the gapped genomic regions in the v1 panel. These refinements make the v2 panel a robust resource for studying cranberry genetics and cranberry breeding.

The microhaplotype database for the cranberry v2 marker panel includes alleles from newly added markers and those retained in the v1 panel, ensuring that any previously validated information is not discarded. A total of 12,821 microhaplotypes from the retained v1 markers were integrated into the latest microhaplotype database. Summary statistics show that most loci have low levels of allelic variation (median = 3 alleles), while the high standard deviation arises from a subset of loci with extreme variability (Table 2). Our BLAST-based classification showed that these loci were dominated by off-target best hit (mapping outside the intended site) or putative paralogs (multiple equally good hits) rather than true alleles at a single locus (Table S2). For example, the most variable marker, chr11_035249665 (448 microhaplotypes), had 419 off-target best hits plus 16 putative paralogs (435/448; 97% putative non-allelic), and chr03_024644041 showed a similar pattern (113/131; 86%). In contrast, many loci exhibited clean single-locus behavior with all microhaplotypes mapping to the intended site and no putative paralogs (e.g., chr07_017980443; 64/64 design matches; chr01_018734260: 36/36; chr07_001562431: 32/32; and chr09_008835521: 28/28). Together, these observations indicated that the large variance in microhaplotype counts is driven by a small number of multi-locus amplifications, while the majority of markers remain locus specific. We flagged these putative paralogous microhaplotypes in the database since they will likely occur in future genotyping results.

**Table 2:** Summary statistics of the number of microhaplotypes per target marker locus for the eight microhaplotype databases

Species	#Loci	Microhaplotype statistics						
		mean	Standard deviation	minimum	25%	50%	75%	maximum
Alfalfa	3000	12	9	2	6	10	15	160
Blueberry	3000	10	11	2	4	7	11	270
Cranberry	3050	5	9	2	2	3	5	448
Cucumber	3059	3	6	2	2	2	4	256
Pecan	3100	8	8	2	5	7	10	177
Potato	3913	9	12	2	4	6	10	335
Strawberry	5000	8	9	2	2	6	10	274
Sweetpotato	3120	12	13	2	6	9	14	180

### Cucumber microhaplotype database

The cucumber microhaplotype database (v10) consists of 9823 microhaplotypes derived from 8272 genotyped samples (Fig. 2). These microhaplotypes originate from 3059 target loci (manuscript in preparation), which are evenly distributed across all seven cucumber chromosomes. The number of alleles per locus had a mean of 3, with a standard deviation of 6, indicating uneven allele diversity across surveyed loci (Table 2). A few DArTag loci exhibited extremely high rates of polymorphism, with a maximum 256 alleles, which likely result from paralogous sequence amplification or hot spot regions of genetic variability.

The cucumber microhaplotype database provides an essential resource for dissecting genomic and allelic diversity in cucumber. The genotyped samples were from several biparental F_1_ and backcross populations, which resulted in the small number of alleles per locus (mean ~3.2). As no diverse collections have been genotyped using the cucumber marker panel, it is not yet feasible to evaluate the genetic diversity across the cucumber genome.

### Potato microhaplotype database

The potato microhaplotype database (v14) is comprised of 35,506 microhaplotypes across 3913 DArTag loci (Fig. 2), distributed across all 12 chromosomes of the potato reference genome DM6.1 (Pham et al. 2020) and a few trait markers that are not located in the DM6.1 genome (Endelman et al. 2024). These microhaplotypes were constructed from data across 3,102 samples. The number of alleles per locus had a mean of 9, with a standard deviation of 12, indicating a wide range of allele diversity across loci (Table 2). Notably, 50% of loci had six alleles (median value). A small number of loci displayed exceptionally high polymorphism rates, with a maximum of 335 alleles, potentially attributable to factors such as paralogous sequence amplification or regions of extraordinarily high genetic variability.

The relatively high mean number of alleles per locus reflects substantial genetic diversity, which is consistent with the structural complexities of the potato genome and its propagation history. While clonal propagation suppresses recombination, it is highly effective at preserving existing diversity within an autotetraploid system. The wide range of allele counts emphasizes the mosaic distribution of diversity, highlighting both highly polymorphic regions and those with more limited variability across the genome. These insights will contribute to breeding programs, genetic mapping studies, and other research applications centered on potato population and genome dynamics.

### Strawberry microhaplotype database

The strawberry microhaplotype database (v3) contains 38,227 microhaplotype sequences derived from 5000 DArTag loci (Fig. 2) distributed across all 28 chromosomes (7 chromosomes with four sub-genomes: A, B, C, and D) (Hardigan et al. 2023). These microhaplotypes were derived from 1880 samples. While 50% of loci exhibited six or fewer alleles (median value), 75% of loci had 10 or fewer alleles (Table 2). The number of alleles per locus had a mean of 8 and a standard deviation of 9, indicating notable variability in allele diversity among loci. The median value 6 indicated a right-skewed distribution driven by a small number of highly polymorphic loci (maximum allele count at 274). Such high allele counts likely result from genetic hot spots, paralogous amplification, or regions of high genetic variability.

### Alfalfa microhaplotype database

The alfalfa microhaplotype database (v50) has reached a notable milestone, containing 35,259 microhaplotypes across 3000 DArTag marker loci (Zhao et al. 2023), averaging ~ 12 alleles per locus. These microhaplotypes were discovered through 25 genotyping projects (> 15,600 samples) conducted by the alfalfa public breeding community, spanning diverse and structured polycross populations and breeding programs. The number of alleles per marker locus ranges from 2 to 160, with extended allele counts potentially signifying highly diverse genomic regions or paralogous amplification (Table 2). Allele diversity per locus is high compared to other species, as indicated by a mean of 12 alleles (SD = 9) and a median of 10 alleles. Approximately 25% of loci had 2 to 6 alleles, while 75% had 15 alleles or less.

The trend in microhaplotype discovery shows rapid growth in earlier database versions, followed by a plateau around ~ 35,000 microhaplotypes despite the continued addition of samples (Fig. 2). This plateau suggests that the current marker set (3000 loci) has effectively captured most genetic diversity at the 3 K target loci among the US alfalfa breeding populations. This plateau may also reflect database saturation, such that most of the *Medicago sativa* alleles existing across these loci have been captured. Notably, while microhaplotype discovery has plateaued, the database serves as a powerful resource for genomic analysis within cultivated alfalfa and its close relatives (Zhao et al. 2024c).

**Fig. 3:**
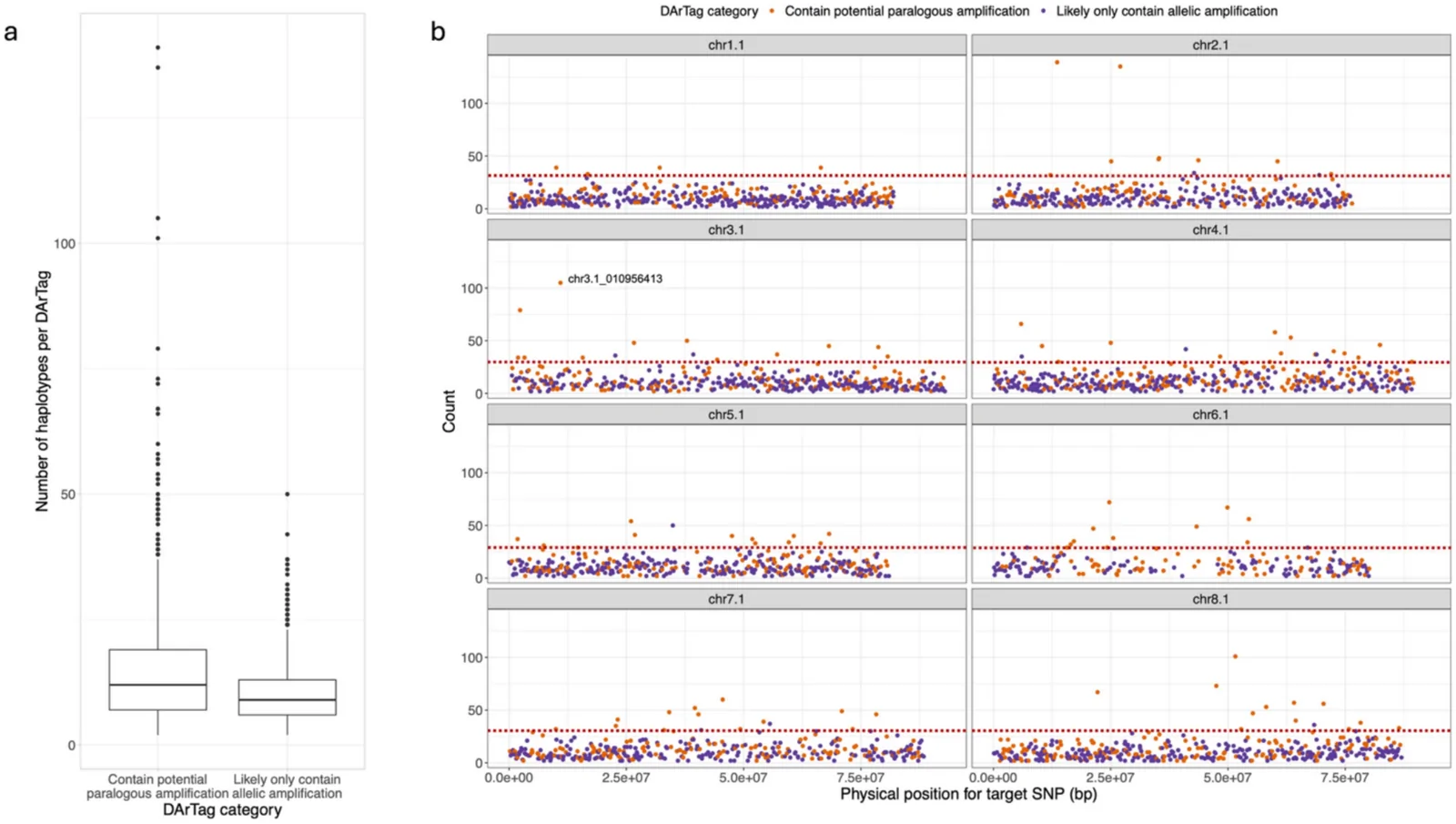
Distribution and genomic position of microhaplotype counts per DArTag locus in alfalfa, grouped by amplification category. **a** Boxplots showing the number of microhaplotypes for DArTag loci containing potential paralogous amplification and those likely containing only allelic amplification. **b** Scatter plots or microhaplotype counts for individual DArTag loci against their physical positions on target chromosomes. The red dashed line is an indicator of 30 haplotypes per locus

As is common in targeted genotyping platforms, not all resulting amplicons originate from the intended genomic locations due to sequence duplications within the genome. In polyploid species, the set of corresponding loci across homologous chromosomes are known as homologous groups (HGs), which share high sequence similarity and derive from the same ancestral sequences. HGs provide a framework for distinguishing true allelic variation from paralogous amplification. To this end, all 35 K microhaplotype sequences were aligned to the four homologous chromosome sets of the alfalfa cultivar ‘XinJiangDaYe’ (Chen et al. 2020). Syntenic regions across homologous chromosomes were used to determine the most likely syntenic alignment if a microhaplotype could be aligned to multiple loci on one chromosome. A total of 22,159 microhaplotypes from 2,314 DArTag loci (Fig. S2 & S3; Table S3) are likely true allelic variants (i.e., match the panel design specification), ranging from 210 in HG6 to 426 in HG4 (Table S3). A total of 1085 DArTag loci contained potential paralogous amplifications and on average, produced more microhaplotypes per locus than the 1915 loci containing only true allelic variants (Fig. 3a). Four DArTag loci with more than 100 microhaplotypes all exhibited paralogous amplifications. For example, locus chr3.1_010956413 was located within a gene (*MS.gene044613*), which encodes a leucine-rich repeat and nucleotide-binding-ARC domain (NBS-LRR or NLR) disease-resistance protein (Fig. 3b). Among the 1915 DArTag loci with no evidence of paralogous amplifications, 12 have more than 30 alleles each (Fig. 3b; Table S3), indicating that these genomic regions possess relatively high genetic diversity and/or are not under strong selection pressure.

For the 1,915 DArTag loci without evidence of paralogous amplification, we further examined their copy numbers, defined here as the number of homologous chromosomes in which each locus is present. Among these loci, 1815 (94.8%) were found in three (21.5%) or all four (73.3%) homologous chromosomes within each of the eight homologous groups, while 73 had two copies and 27 loci had only one copy (Fig. S4). Although alfalfa is tetraploid, the observed variation in copy numbers across the genome underscores the complexity of its genomic structure. Additionally, loci with fewer copies (less than 4) are likely due, or at least in part, to incomplete genome assembly rather than true biological absence.

### Blueberry microhaplotype database

The blueberry microhaplotype database (v20) is comprised of a total of 28,653 sequences generated from 95 genotyping plates (~ 8930 samples) (Fig. 2). It represents a comprehensive characterization of genetic diversity across 3000 target loci (Zhao et al. 2024b), with a genomic insert size of about 54 bp (Table 2), evenly distributed on all 12 chromosomes. The number of microhaplotypes per locus demonstrated a mean of ~ 10 alleles, with a high variability indicated by a standard deviation of 11. Quartile statistics indicated that more than 50% of the loci contained 7 alleles or fewer. The minimum number of microhaplotypes per locus was two, while the maximum recorded was 270 alleles, showcasing a significant range in polymorphism across loci or paralogous amplifications for some loci.

To characterize microhaplotypes potentially resulting from allelic vs. paralogous amplifications, all 28 K microhaplotype sequences were aligned to the four sets of homologous chromosome sequences of the tetraploid blueberry cultivar ‘Draper’. Syntenic regions across homologous chromosomes were used to identify the most probable syntenic alignment when a microhaplotype matched multiple loci on the same chromosome. In total, 18,031 microhaplotypes from 2535 DArTag loci (Figs. S5 & S6; Table S4) were identified as true allelic variants. Across all 3000 loci, 1053 with potential paralogous amplifications tended to produce more haplotypes per locus than the 1947 loci containing only allelic variants (Fig. S7). Among the 1947 loci without evidence of paralogous amplification, seven had more than 30 alleles each (Fig. S7b; Table S4), with Chr03_005753680 and Chr07_033695728 having 82 and 81 alleles, respectively, followed by Chr05_003355218 with 52 allele (Fig. S7b).

Copy numbers of the 1947 loci without paralogous amplification were further analyzed. Of these, 1459 (74.9%) had three (31.3%) or four (43.7%) copies across the 12 homologous groups, whereas 264 loci had two copies and 224 had only one copy (Fig. S8). Notably, of the 224 loci with a single copy in the reference genome, 45 (20.1%) and 67 (29.9%) were located on chromosomes 4 (VaccDscaff6) and 12 (VaccDscaff22), respectively, which agrees with previous discovery that large genomic regions on these two chromosomes lack synteny (Colle et al. 2019). In addition, 66 out of 146 DArTag loci designed on chromosome 10 (VaccDscaff20) had three copies, reflecting that the distal region of chromosome 10 (VaccDscaff20) shares synteny with chromosomes 22 (VaccDscaff28) and 34 (VaccDscaff44), but not with chromosome 46 (VaccDscaff48) (Zhao et al. 2024a), while 52 of these 146 DArTag loci had four copies with three of them in HG10 but one copy in HG6 (chromosome 30/VaccDscaff19).

### Pecan microhaplotype database

The pecan microhaplotype database (v9) is comprised of 26,073 alleles from 3100 target loci (Chen et al. 2026), derived from 6768 genotyped samples (Fig. 2). The loci are evenly distributed across 16 chromosomes, and each of the 3100 loci shows diverse allele counts. Most loci exhibit moderate levels of allelic diversity, with half of the loci containing seven or fewer alleles (Table 2). A small number of loci with extremely high allele counts may arise due to paralogous sequence amplification or hot spots of genetic variation.

The complete pecan linkage map (Chen et al. 2026) shows no large-scale structural variation (e.g., homologous sequence rearrangements) between the studied F_1_ population and the reference genome. To compare the performance between microhaplotypes and SNPs, linkage maps were constructed for each of the three marker datasets: microhaplotypes, target-SNPs-dataset, and all-SNPs-dataset (target plus off-target SNPs).

**Fig. 4:**
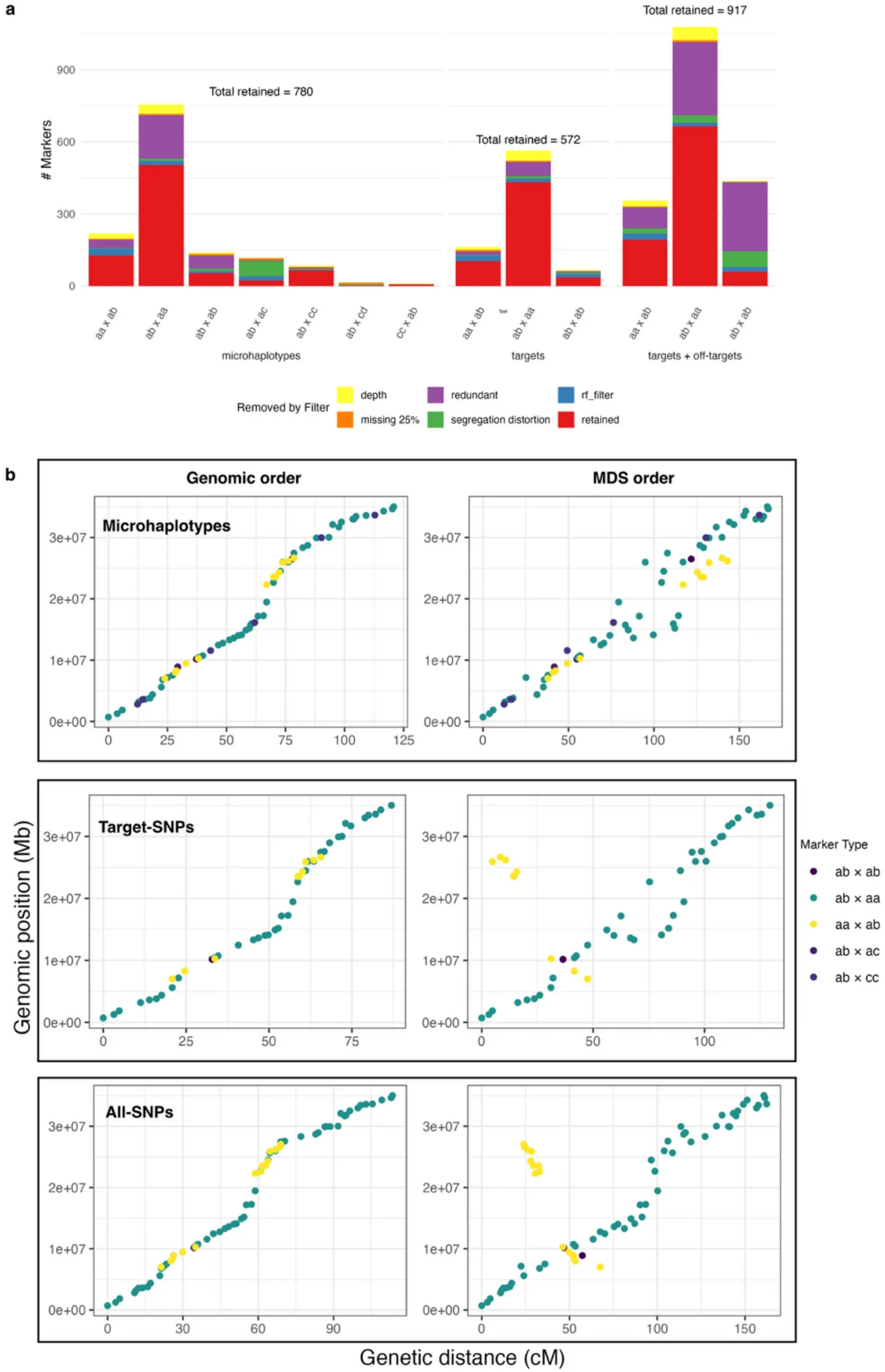
Comparison of marker ordering and filtering outcomes across three marker datasets (microhaplotypes, target-SNPs-dataset, all-SNPs-dataset). **a** Summary of marker filtering by genotype class across the three datasets. Bars represent the number of markers per genotype class removed by specific filters. **b** Linkage marker ordering for chromosome 8 using genome position (left panels) and multidimensional scaling (MDS) genetic distance (right panels)

Filtering statistics further highlighted key differences among the three marker sets (Fig. 4a, Table S5). The all-SNPs-dataset showed a high number of loci removed by the redundancy filter, which is expected given their proximity to target markers (within 81 bp). The microhaplotype dataset retained a larger number of markers capable of tracking recombination events and providing information for both parents’ meiosis. In contrast, the target-SNPs-dataset and all-SNPs-dataset contained mostly ab × aa markers (single-parent informative) and very few ab × ab markers (informative for both parents) after filtering. These patterns were consistent across all 16 chromosomes, with chromosome 8 being the most prominent (Figs. 4b and S9). Therefore, in the target-SNPs-dataset and all-SNPs-dataset, the aa × ab markers were frequently ordered independently and placed far from their expected genomic positions using the MDS method since not enough informative markers for both parents (heterozygous × heterozygous) were present to relate single-parent informative markers (aa × ab and ab × aa).

The all-SNPs-dataset showed a greater number of gaps when linkage maps were ordered according to the reference genome, indicating a higher incidence of genotyping errors among the retained markers. In contrast, target-SNPs-dataset demonstrated fewer gaps in the reference-based linkage map, suggesting lower genotyping error rates compared to the all-SNPs-dataset. However, it contained relatively few markers informative for both parents, which impaired ordering performance using MDS algorithm. The microhaplotype dataset retained more loci throughout the filtering pipeline, had fewer gaps, and preserved a higher proportion of markers informative for both parents. These attributes contributed to more robust ordering when applying the MDS algorithm.

### Sweetpotato microhaplotype database

The sweetpotato DArTag panel is a key molecular tool that was developed and applied in various collaborative projects to advance sweetpotato (2n = 6x = 90) genetics and breeding worldwide (Zhao et al. 2024a). The latest version of the associated microhaplotype database (v19) consists of 37,593 sequences across 3120 genomic loci, which are evenly distributed across the 15 chromosomes (Fig. 2). These microhaplotypes were derived from genotyping data of 9212 individual samples. The number of alleles per locus ranges from 2 (minimum) to 180 (maximum), with an average of 12 alleles per locus (Table 2). The median number of alleles per locus is 9, and the interquartile ranges between 6 (25th percentile) and 14 (75th percentile). The wide distribution of alleles suggests the markers effectively capture broad genetic variations.

**Fig. 5:**
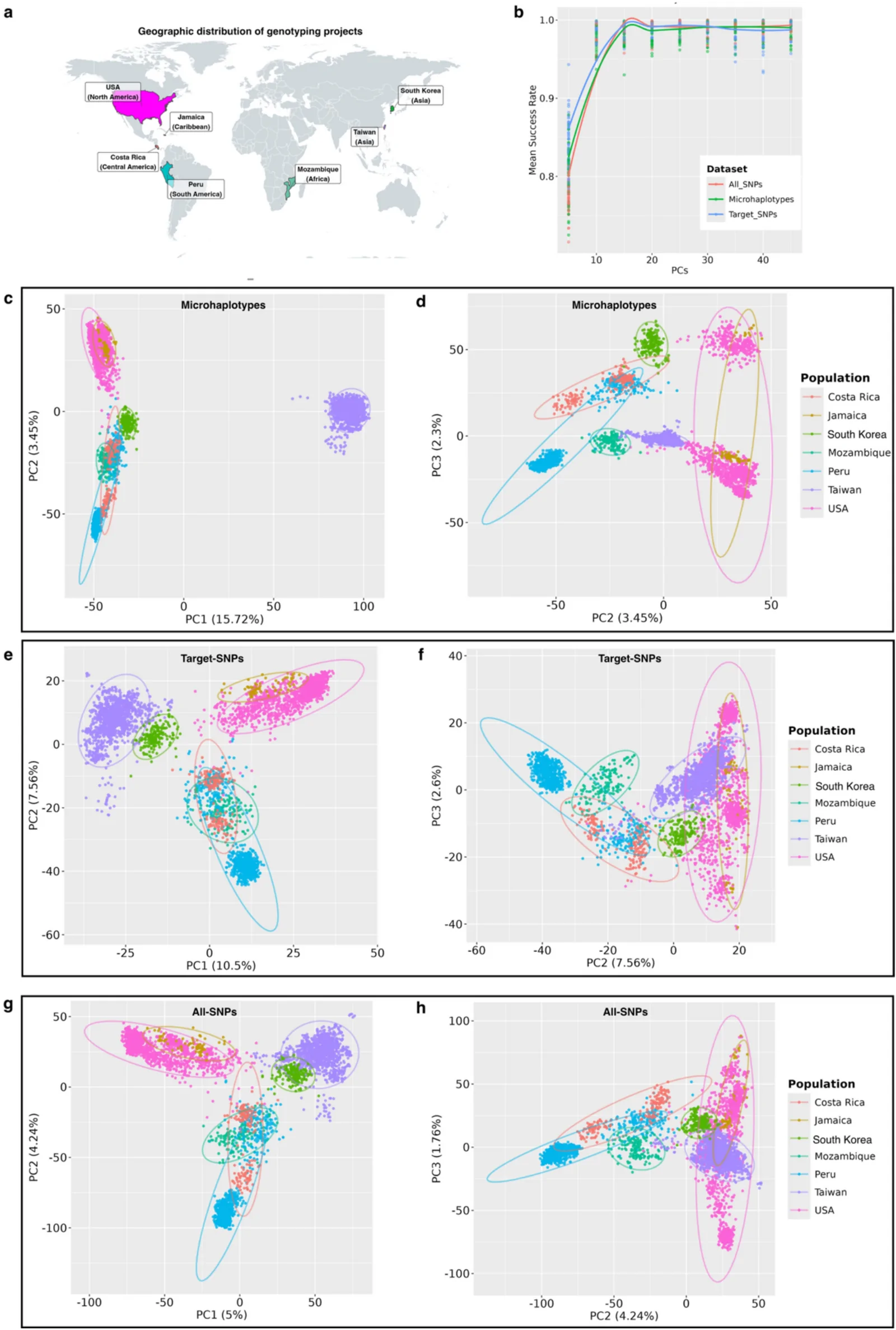
Geographic context, cross validation accuracy and principal component analysis (PCA) of microhaplotypes and SNP markers in sweetpotato. **a** Geographic distribution of genotyping projects using the sweetpotato 3 K DArTag panel. **b** Discriminant analysis of principal components (DAPC) cross-validation accuracies using 5–50 PCs for microhaplotypes, target-SNPs-dataset, and all-SNPs-dataset. **c**, **d** PCA plots for PC1 vs. PC2 (**c**) and PC2 vs. PC3 (**d**) of the microhaplotype dataset. **e**, **f** PCA plots for PC1 vs. PC2 (**e**) and PC2 vs. PC3 (**f**) of the target-SNPs dataset. **g**, **h** PCA plots for PC1 vs. PC2 (**g**) and PC2 vs. PC3 (**h**) of the all-SNPs-dataset

The versatility and efficacy of the sweetpotato marker panel are evidenced by its extensive adoption across breeding programs from multiple continents. Participating programs include those in the USA, Mozambique (Africa), Korea and Taiwan (Asia), Peru (South America), Jamaica (Central America), and Costa Rica (Caribbean/Central America) (Fig. 5A).

We evaluated the performance of three marker sets on population genetics inference in sweetpotato: microhaplotypes, target-SNPs-dataset, and all-SNPs-dataset (target + off-target). To ensure high-quality genotype data while retaining informative low-frequency and private alleles, we implemented a population-aware filtering strategy in which a microhaplotype was retained if it met either a global missingness threshold of ≤ 20% across all samples, or a per-population missingness threshold of ≤ 20% in at least one population. After filtering for marker missingness and alternative allele frequency (AAF) of > 0.05, a total of 2772 (27,220 microhaplotypes) out of 3119 marker loci (28,338 microhaplotypes) and 4087 samples with < 20% missing data were kept. The target-SNPs-dataset included the target SNPs present within the 2772 retained loci. The same filters were applied to the target-SNPs-dataset, resulting in 2534 SNPs. The all-SNPs-dataset are comprised of all SNPs in the 2772 marker loci, resulting in a total of 21,002 SNPs across 4087 samples. After removing monomorphic sites in all-SNPs, 20,935 markers remained. Because these SNPs were extracted directly from the retained microhaplotypes, no additional filtering was applied beyond removal of monomorphic sites.

To evaluate how well the retained panel captures population-specific variation, we enumerated project-specific microhaplotypes among the retained loci (Table 3). Across the seven projects, we identified 8711 project-specific microhaplotypes, spanning 3288 marker-project instances in which at least one private microhaplotype occurred. It should be noted that the distribution of these private variants is heavily shaped by highly uneven sample sizes, ranging from 72 samples (Jamaica) to 1352 samples (Taiwan). Because allele discovery scales non-linearly according to a standard rarefaction curve, larger sample cohorts possess vastly higher statistical power to capture rare, low-frequency variants. Because of this rarefaction effect, estimates of allele richness in smaller populations are highly sensitive to stochastic sampling effects. This technical phenomenon is clearly illustrated when comparing two cohorts with nearly identical sample sizes: the USA cohort (*N* = 1358) and the Taiwan cohort (*N* = 1352). Despite their equivalent sample scales, the Taiwan cohort revealed a vastly higher raw count of private microhaplotypes (7340 alleles across 2333 markers) compared to the USA cohort (367 alleles across 308 markers). This pronounced disparity reflects differences in the underlying architecture of their respective gene pools rather than a sampling artifact.

**Table 3:** Project-level private microhaplotypes and marker information in sweetpotato

Project/region	Sample size (N)	#Private_microhaplotypes	#Markers with private microhaplotypes
Costa Rica	165	261	140
Jamaica	72	2	2
Mozambique	155	27	27
Peru	794	274	214
South Korea	191	440	364
Taiwan	1352	7340	2233
USA	1358	367	308

Our comparative analysis revealed that microhaplotypes consistently provided a more efficient and stable representation of population structure than either target-SNPs-dataset or all-SNPs-dataset. Principal component analysis (PCA) revealed marked differences in the resolution of population structure (Fig. 5c–h). In PC1-PC2 space, the microhaplotype PCA displayed visibly tighter clusters for each population with minimal overlaps, with Taiwan strongly differentiated along PC1 and subtler separations among other populations along PC2. In contrast, the SNP-based PCA exhibited less distinct separation with noticeable overlap among several populations, particularly Costa Rica, Mozambique, South Korea, and Taiwan. Similar trends were observed in PC2-PC3 space. The variance explained by the leading PCs further highlighted these differences. For microhaplotypes, PC1, PC2, and PC3 explained 15.72%, 3.45%, and 2.3% of the total variance, respectively, compared to 10.5%, 7.56%, and 2.6% for target-SNPs-dataset, and 5%, 4.24%, and 1.76% for all-SNPs-dataset. Therefore, PC1 of the microhaplotypes captured more variance compared with the SNP equivalents, suggesting stronger separation along the most informative axis.

Discriminant analysis of principal components (DAPC) further highlighted the discriminatory advantage of microhaplotypes (Table 4). Cross-validation showed that 86.3% of between-group variance was captured by the first linear discriminant axis (LD1) for microhaplotypes compared with approximately 60% for both SNP sets, an improvement of over 25%. The LD1 eigenvalue was correspondingly larger for microhaplotypes (854,002) than for all-SNPs-dataset (215,300), and target-SNPs-dataset (112,056), reflecting tighter cluster resolution. Despite these differences in dimensional efficiency, all three marker types achieved very high classification accuracies (mean success rate > 98%; ANOVA: F = 1.495, *p* = 0.225) (Fig. 5b). At low dimensionality (5 PCs), targeted-SNPs-dataset achieved the highest early success rate (84.3%), followed by microhaplotypes (80.2%) and all-SNPs-dataset (77.7%), likely reflecting the pre-selected informativeness of target SNPs. As the number of retained PCs increased, target-SNPs-dataset accuracy degraded starting around 40 PCs, whereas microhaplotypes maintained a stable high-success plateau near 99%, suggesting greater robustness to model complexity.

**Table 4:** Eigenvalue distribution and proportion of variance explained by the three marker sets and linear discriminant (LD) axis in sweetpotato populations

	eigenvalue			Fractions		
Axis	Microhaplotypes	All_SNPs-dataset	Target_SNPs-dataset	Microhaplotypes	All_SNPs-dataset	Target_SNPs-dataset
LD1	854,002	215,300	112,056	86.26	60.61	60.28
LD2	74,596	89,897	47,587	7.53	25.31	25.6
LD3	41,989	35,015	16,007	4.24	9.86	8.61
LD4	16,736	12,488	8395	1.69	3.52	4.52
LD5	1921	1731	1237	0.19	0.49	0.67
LD6	805	784	618	0.08	0.22	0.33

### Comparative insights across all eight databases

Microhaplotypes are sets of closely linked SNP sites within a small region of the genome (assumed not to recombine), making them a powerful tool for genotyping and genetic diversity studies. The development of these microhaplotype databases across multiple crops has enabled the discovery of rich allelic diversity while providing the plant germplasm, breeding, and genetics community with resources to dissect functional diversity, population structure, and trait architecture using cost-effective targeted genotyping. However, direct cross-species comparisons of raw allele metrics or database growth curves must be treated with significant caution. These eight crops differ dramatically for many confounding biological factors such as genome size, ploidy, reproductive methods, natural population sizes, selection bottlenecks, and the cumulative artificial selection process to create the crops we see today. In addition to biological differences, there are intrinsic ascertainment biases that impact the databases which were shaped by the markers chosen for the panels, the samples chosen for genotyping, and the collaborators willing to share data. The observed differences in alleles per locus and total database size, therefore, reflect technical panel attributes and ascertainment effects as much as true baseline biology between species.

The microhaplotype databases for these eight species were developed with varying numbers of genotyped samples (Table 1). Factors such as the scale of genotyping, species-specific genome complexity, marker diversity, and the genomic insert size used during genotyping library preparation impacted the resulting resources. Unsurprisingly, longer genomic insert sizes (e.g., 81 bp) tend to capture more variant sites compared to shorter insert sizes (e.g., 54 bp), allowing for more variants to be detected per amplicon locus. For example, species like sweetpotato and pecan, which utilize 81 bp panels, show substantial allele diversity relative to their genomic complexity, partly because of the longer genomic region available for detecting polymorphisms.

All eight microhaplotype databases were developed by following a standardized pipeline that includes stringent ID conventions, filtering, and sequential updates to maintain biological relevance. Notably, the databases include both orthologous (i.e., microhaplotypes originated from conserved loci between divergent species) and paralogous (i.e., related loci within a genome, product of duplication) microhaplotypes, the latter of which are more common in complex genomes with higher levels of duplications. The retention of paralogous alleles may seem counterintuitive at first glance, since they cannot be used in breeding. However, because these alleles are likely to be encountered in future genotyping experiments, it was important to codify them and append a confidence rating that indicates to the users that they are likely paralogous alleles.

Among the databases, alfalfa (autotetraploid), sweetpotato (hexaploid), and blueberry (autotetraploid) exhibit the highest genetic diversity, with average allele counts per locus of 12, 12, and 10, respectively. The polyploid nature of these crops likely contributes to their exceptional allelic richness. Furthermore, these three species also represent the largest genotyping scales undertaken on the respective DArTag panels. The alfalfa database, based on 167 plates of genotyped samples (over 15,600 samples), stands out as the most extensive. Alfalfa’s saturation plateau (~ 35,000 microhaplotypes) demonstrates that the high genotyping scale successfully captures most genetic diversity at the 3 K target loci within the US breeding population. Genotyping additional wild *Medicago* accessions and international breeding lines could reveal additional diversity. For sweetpotato, the global importance, spanning Africa, Asia, and the Americas, is mirrored in the diversity captured in its database, which encompasses samples from 9212 samples and aligns with a worldwide population structure.

In contrast, the compact diversity observed in the cucumber database represents an illustrative example of narrow sampling scale rather than definitive biological uniformity per se. A mean of 3 alleles per locus and 75% of loci containing no more than 4 alleles likely reflects narrow germplasm sampling from a single active breeding program and/or the limited genetic diversity inherent in cucumber relative to the other species analyzed. Consistent with this, prior work indicates that expanding this panel’s testing on wild, sexually compatible relative populations would likely broaden its descriptive allelic base (Zhang et al. 2017; Abdel-Salam et al. 2020; Tirnaz et al. 2022). On the other hand, the octoploid strawberry database reveals rich allele diversity with a mean of 8, despite a relatively smaller genotyping scale (20 plates). The high allelic diversity evidenced here traces back to the selection of sub-genome-specific loci to isolate functional diploid variation across a complex allo-octoploidy background, capturing deep ancestral polymorphisms within a small, targeted marker pool (Hardigan et al. 2023). Additionally, the inclusion of various species, subspecies, forms, and hybrids within the genus *Fragaria* led to the allelic enrichment.

### Data accessibility and community engagement

In alignment with the FAIR principles (https://www.go-fair.org/fair-principles/), all microhaplotype sequences will be made publicly available. The allele data, metadata (including project and principal investigator (PI) information, and associated documentation will be hosted through globally accessible and FAIR-compliant platforms, HapApp (https://github.com:Breeding-Insight/HapApp_utils) and HapSearch (under development). Users who are interested in obtaining samples or germplasm linked to specific alleles must follow established access procedures. Interested users will need to contact the relevant PIs and adhere to the AgCommons Framework (https://agdatacommons.nal.usda.gov/), or a similar standard protocol for germplasm access. The PI has the right to share the resources while respecting intellectual property rights, mutually agreed terms, and proper documentation.

## Discussion

The eight curated microhaplotype databases developed here provide a standardized framework for analyzing genetic diversity and assisting breeding programs across the surveyed crops. Through consistent microhaplotype ID conventions, filtering pipelines, and sequential updates, these resources capture both orthologous and documented paralogous microhaplotypes, ensuring broad relevance to future genotyping. Microhaplotypes deliver richer allelic diversity and greater resolution than biallelic SNPs, a benefit especially evident in complex, polyploid genomes such as alfalfa, blueberry, and sweetpotato. Our methodological ‘stress tests’ in pecan and sweetpotato revealed the operational value of the increased informativeness, facilitating linkage map ordering, and resolving fine-scale population structure under specific experimental contexts.

### Methodological filtering and project-scale considerations

When processing a new genotyping report, the first safeguard filter to avoid technical artifacts (e.g., PCR and sequencing errors) is the frequency of the microhaplotypes at the project level. We required an allele to be present in a minimum of 10 samples or 5% of total samples (whichever is smaller) in order for a microhaplotype to be considered valid candidate. In practice, because individual projects processed through the pipeline typically scale from 2 to 20 96-well plates (approximately 188 to 1,880 samples with each plate having two control wells), this mathematical configuration defaults to a fixed threshold of 10 samples for the vast majority of analyzed experiments.

While a flat proportional threshold (e.g., 5% of total samples) is standard within small, localized mapping populations, its uniform implementation across large-scale projects introduces a significant ascertainment bias. Specifically, a proportional frequency filter would require rare variants to appear in 94 samples for a 20-plate project to avoid deletion and disproportionately purging rare variations as project scales increase. Utilizing a low absolute floor at the project level ensures the microhaplotype database remains comprehensive by safeguarding rare alleles for future trait discovery and germplasm tracking. Users can easily apply strict, population-specific proportional filters (such as minor allele frequency) appropriate for their downstream specific analytical requirements.

### Orthologous and paralogous microhaplotypes

While most loci exhibited low to moderate microhaplotype counts, the extremely high allele counts observed at some loci are likely due to paralogous sequence amplification, in addition to natural orthologous genetic diversity. Paralogous sequences arise from duplicated regions within the genome, which can be unintentionally captured and amplified during PCR amplification due to sequence similarity. These loci can be important for identifying hot spots of variation, but they may also reflect technical artifacts rather than true orthologous diversity.

Comparative patterns in alfalfa and blueberry microhaplotypes illustrated how genome structure shapes these metrics. Though both species have a similar number of loci without paralogous amplification (1915 and 1947 loci, respectively), their copy-number distributions differ (Tables S3 & S4, Figs. S4 & S8). In alfalfa, 94.8% of the non-paralogous loci occur in three or four copies (Fig. S4), matching the expected dosage for a largely autotetraploid species. In contrast, only 74.9% of blueberry loci fall into this range. Instead, blueberry shows substantially higher proportions of single-copy (20.1%) and two-copy (29.9%) loci (Fig. S8). This elevated number of reduced-copy loci in blueberry suggests more extensive post-whole-genome duplication structural modifications than that observed in alfalfa. These patterns are consistent with current evidence that highbush blueberry originated through a complex polyploidization process involving hybridization among multiple diploid *Vaccinium* species followed by genome duplication and subsequent structural divergence (Colle et al. 2019; Mengist et al. 2023). The cultivar ‘Draper’ is predominantly *V. corymbosum* with minor introgressions (< 5%) from *V. tenellum*, *V. ashei*, and *V. darrowii*, which may help explain part of the observed pattern (Hancock 2004). The unique inter-chromosome translocation between chromosomes 6 and 10 could affect pairing and recombination of these two chromosomes in ‘Draper’ but was not observed in a wild diploid relative ‘W85’ (Mengist et al. 2023) or reported in other tetraploid highbush blueberries.

Most repetitive sequences in the alfalfa ‘XinJiangDaYe’ reference genome are composed of long terminal repeat (LTR) retrotransposons, consistent with previous observations in other legume species (Chen et al. 2020). However, because DArTag markers are predominantly designed within genic regions, most cases of paralogous amplification in our dataset are not likely attributed to LTR-derived repeats. Notably, we identified a highly paralogous marker (chr3.1_010956413) with 105 microhaplotypes (rank of 3) located within *MS.gene044613*, an NLR disease resistance protein. This observation aligns with earlier findings in *M. truncatula*, a wild relative of cultivated alfalfa, in which a dense cluster of NLR genes was reported on chromosome 3.1 (Ameline-Torregrosa et al. 2008). Together, these results suggest that local gene family expansion could play a role in generating paralogous signals in DArTag genic markers.

### Linkage map performance and phasing considerations

Comparison of linkage maps created using microhaplotypes vs. SNPs highlighted the advantages of microhaplotypes in providing higher information content and reducing ambiguity in phase estimation (Fig. 4). Because target and off-target SNPs fall within a 54–81 bp read, variants within each amplicon are physically linked and phased directly from reads, making recombination within an amplicon effectively negligible. To ensure a methodologically equitable comparison among datasets, the redundancy filter applied across all datasets was strictly LD-based. Markers exhibiting an identical genotype pattern across every individual were grouped into a shared LD block, and only a single representative marker maximizing data completeness was retained. Under this parameter, the all-SNPs-dataset suffered the greatest reduction in marker density because adjacent off-target SNPs frequently fell into identical LD blocks as their primary target sites, causing them to collapse into a single representative variant. Despite this equitable filtering, the remaining all-SNPs-dataset markers performed poorly in map ordering mainly because they were biallelic. With only two alleles, standard segregation filters lack sufficient statistical power to detect and discard hidden paralogous amplifications. These false-positive SNPs are more likely to persist through traditional filtration steps and ultimately degrade map order. In contrast, the multi-allelic nature of microhaplotypes naturally exposes multi-locus amplifications, providing an inherent biological filter against structural duplication noise while increasing the frequency of markers heterozygous in both parents (e.g., ab × ab). This strengthens linkages among single-dose variant configurations (ab × aa and aa × ab), whereas biallelic datasets rely on rare cross-configurations to bridge parental maps, limiting integration and overall connectivity. Consequently, both target-SNPs-dataset and all-SNPs-dataset (target plus off-target) performed poorly during marker ordering due to the limited number of markers informative for recombination in both parents.

Problematic markers can be filtered out by increasing the stringency of the rf_filter parameter, but good-performing markers can also be removed, especially in datasets with few multi-allelic or highly informative markers. In contrast, the microhaplotype strategy integrates off-target information into multi-allelic markers, which reduces redundancy and enhances the effectiveness of downstream filtering. This highlights the need for careful parameter tuning, especially in datasets with fewer multi-allelic or highly informative markers.

Microhaplotypes also reduce ambiguity in phase estimation. For multi-allelic configurations such as ab × cd, the parental origin of progeny alleles is directly observable, decreasing the reliance on expectation–maximization (EM) inference and lowering the probability of phasing errors. In contrast, biallelic datasets contain ambiguous configurations (e.g., ab × ab) requiring EM inference, increasing the risk of incorrect phasing.

### Resolution of population structure

The high informativeness of multi-allelic microhaplotype genotypes underpin their growing adoption not only in human forensic and ancestry inference applications but also in the plant genetics and breeding realm. The comparative analysis of microhaplotype and SNP data analyzed independently in hexaploid sweetpotato revealed notable differences in allelic diversity, variance captured, and population resolution. The resulting extra diversity using microhaplotypes means that patterns differentiating populations are stronger per locus, which is consistent with findings from other systems. In a study of 28 populations, Medina et al. (2025) revealed significant population structure based on geographical origin, higher genetic diversity values compared to traditional SNP markers, excess heterozygosity (negative FIS values) in 27 out of 28 populations, and minimal inbreeding in founding populations (Medina et al. 2025).

In human genetics, highly polymorphic microhaplotype panels have demonstrated strong performance in relationship detection. For example, a set of 54 microhaplotypes demonstrated high reliability for first-degree relationship detection, approaching the performance of an established forensic panel comprising 27 short tandem repeats (STRs) plus 94 SNPs (Wu et al. 2021). Complementary evidence from ancestry inference research identified 120,000 microhaplotypes from nearly one million SNPs and across multiple benchmarks, microhaplotype subsets consistently outperformed SNP panels in resolving ancestry, particularly in complex or admixed populations (Turchi et al. 2026). Taken together, these cross-disciplinary insights point toward the value of fully utilizing microhaplotypes in plant breeding, particularly highly complexed polyploid species. These advantages are directly relevant for dosage-aware genomic prediction in polyploids. By encoding each microhaplotype as a copy-number dosage (0–2 for diploids; 0–4 for tetraploids, etc.), multi-allelic loci provide richer predictors than biallelic SNPs, capturing local microhaplotype effects and within-locus interactions. Such dosages can be used to build microhaplotype-based relationship matrices for GBLUP or included as regressors in Bayesian whole-genome model to capture more additive variance and reduce phase-related ambiguity compared with individual SNPs.

From a methodological perspective, the implementation of a population-aware filtering strategy addresses a challenge in population genetics by ensuring high data quality without discarding private alleles. This method successfully preserved genetic signals relevant for local adaptation, breeding history, and germplasm differentiation even when these microhaplotypes were scarce across the global dataset. One advantage of applying this strategy is the preservation of thousands of private microhaplotypes in the Taiwan sweetpotato population, which allowed its clear separation from other populations. The distinct clustering of Taiwanese accessions (*N* = 1352) in the PCA, alongside the high volume of private microhaplotypes compared to the similarly sized USA cohort (*N* = 1358), points to the steep trajectory of its underlying rarefaction curve. Rather than indicating a failure of rarefaction principles, this pronounced disparity highlights differences in the underlying architecture of their respective genetic pools. This extensive variation within Taiwanese cohort reflects its breeding history, e.g., repeated cycles of crossing and selection within local breeding programs, frequent use of shared founder lines, region-specific breeding objectives, and relatively limited germplasm exchange with other regions. While this provides an extensive data asset for tracking localized breeding selection and shared founder lines within that specific region, direct biological comparisons of allelic richness against smaller cohorts (such as Jamaica or Mozambique) are restricted by non-linear rarefaction effects. This underscores the importance of interpreting database growth curves and private variant totals as metrics of operational cohort/repository completeness and sampling scale rather than absolute evolutionary baselines.

### Scope, limitations, and methodological considerations

Another important application of microhaplotypes is for curation and management of Plant Genetic Resource (PGR) collections. They can be used to: (i) resolve population structure and quantify diversity within and among collections; (ii) identify redundancy and gaps to optimize regeneration, safety-duplication, and sampling strategies; (iii) verify accession identity and detect mixtures or mislabeling to improve passport data and taxonomic assignments; and (iv) define representative core subsets for evaluation and pre-breeding. The standardized IDs and cross-project comparability enable genebanks to benchmark collections across repositories and over time, directly supporting trait discovery and the deployment of novel variation into breeding pipelines.

It is also critical to note that the utility of these microhaplotype panels is context-dependent. Because the underlying DArTag assays target genic regions, the diversity captured is representative of functional genomic spaces rather than a random, genome-wide sampling. Furthermore, while we did not systematically validate population structure and linkage mapping across all eight species in this study, we targeted highly challenging outcrossing, heterozygous, or autopolyploidy species (such as pecan and sweetpotato) as proof-of-concept case studies. While the exact magnitude of these improvements may not replicate identically across all species, populations, or experimental designs, these workflows demonstrate a reproducible and highly stable framework that captures local haplotype phase, offering a clear methodological advancement over traditional biallelic SNP analyses.

### Implications of microhaplotype analysis and future directions

Looking forward, the integration of microhaplotypes into both basic and applied research has the potential to markedly improve analytical efficiency and resolution. The efficiency of microhaplotype-based analyses in low-dimensional space makes them particularly attractive for targeted genotyping in resource-limited breeding programs, where computational capacity and sequencing resources are constrained. At present, relatively few tools can fully accommodate multi-allelic data, particularly in complex polyploid species (Table 5). Expanding the availability and performance of software that can handle multi-allelic datasets in both diploid and polyploid contexts will greatly accelerate the integration of microhaplotypes into breeding decision-making pipelines. Such tool development will enable breeders and researchers to fully leverage the linkage phase information and high allelic diversity inherent to microhaplotype markers.

**Table 5:** List of computational tools available with multi-allelic marker support

Tool name^a^	Analysis	Support polyploids	Citation
Polysat	Population diversity	Yes	(Clark and Jasieniuk 2011; Clark and Schreier 2017)
PLINK 2.0^b^	HWE, LD, Relationship matrix, GWAS	No	(Chang et al. 2015)
polyRAD	Genotype calling	Yes	(Clark et al. 2019)
RAINBOWR	GWAS	No	(Hamazaki and Iwata 2020)
mpQTL	GWAS	Yes	(Thérèse Navarro et al. 2022)
OneMap	Linkage mapping	No	(Taniguti et al. 2022)
ADAM-multi	Population simulation	Yes	(Chu and Jensen 2025)

^a^All described tools also support highly heterozygous species

^b^As of January 10, 2026, release

The current framework is designed to be species-agnostic and expandable for DArTag genotyping panels. With HapApp, users can create, update, and maintain species-specific microhaplotype databases for taxa not included in this study by processing MADC files, assigning fixed microhaplotype IDs, and producing updated databases. To make the microhaplotype databases more amenable, we are actively developing a user-friendly platform, HapSearch, which could serve as an interactive bridge between raw genomic information and practical breeding decisions. With HapSearch, researchers and breeders can rapidly locate, evaluate, and act upon relevant allelic variations captured in the databases. The envisioned features of HapSearch would enhance the value of microhaplotype resources for plant breeding and genetics. First, the system will allow targeted mining of alleles of interest by crop species, sequence similarity, or project-specific keywords. Second, integrated filtering functions will enable users to narrow search results to specific locus. Multiple sequence alignment will reveal all existing variations for a locus and allow users to identify accessions carrying unique microhaplotype alleles. Third, to promote collaboration and resource exchange, the platform could provide access to PI or germplasm collection curator contact information, enabling rapid follow-up for germplasm acquisition or data verification. The suite of platforms from processing and maintaining microhaplotype databases to user-friendly system for mining microhaplotypes will greatly accelerate the translation of genotypic diversity into practical genetic gain.

## Conclusion

We present standardized microhaplotype databases for eight crops, a species‑agnostic, reproducible pipeline, and the no‑code HapApp software for assigning fixed IDs and updating records from the DArTag genotyping platform. While the current pipeline and HapApp framework is engineered specifically around the vendor-supplied MADC reports, the underlying database structure is conceptually adaptable to other targeted genotyping systems (e.g., GT-seq, AgriSeq, FlexSeq). Because these alternative platforms typically deliver data as raw FASTQ files or VCF variants, secondary preprocessing pipelines are required to extract and reconstruct read-based microhaplotype matrices. Developing these cross-platform format conversion bridges represents a key objective for our software ecosystem’s future development roadmap. These eight microhaplotype databases capture extensive allelic diversity, including orthologous and paralogous signals, and provide higher resolution than SNPs for population structure, linkage mapping, and genotype–phenotype analyses. By retaining more informative markers and reducing phasing ambiguity, microhaplotypes are especially powerful in polyploid and highly heterozygous crops and remain efficient for resource‑limited programs. The databases are FAIR and extensible; community updates and the forthcoming HapSearch interface will make allele mining and cross‑project comparisons routine, strengthening PGR curation and accelerating genetic gain across crops.

## Supplementary Information

Below is the link to the electronic supplementary material.

Supplementary Figure 1

Supplementary Figure 2

Supplementary Figure 3

Supplementary Figure 4

Supplementary Figure 5

Supplementary Figure 6

Supplementary Figure 7

Supplementary Figure 8

Supplementary Figure 9

Supplementary Table 1

Supplementary Table 2

Supplmentary Table 3

Supplementary Table 4

Supplementary Table 5

## Data Availability

Scripts and pipeline for building microhaplotype databases are deposited at GitHub: https://github.com/Breeding-Insight/HapApp_utils; https://github.com/Breeding-Insight/HapApp. The microhaplotype database files of the eight crop species can be downloaded from Zenodo https://doi.org/10.5281/zenodo.19338534.
