# Supplementary material for: Standardized microhaplotype databases and frameworks for assessing and mining crop genetic diversity
Source: Theor Appl Genet. 2026 Aug 25;139(9):244. doi: 10.1007/s00122-026-05340-4 (PMC13506570; doi:10.1007/s00122-026-05340-4)
Supplement: Supplementary file 4 [file 122_2026_5340_MOESM5_ESM.pdf]

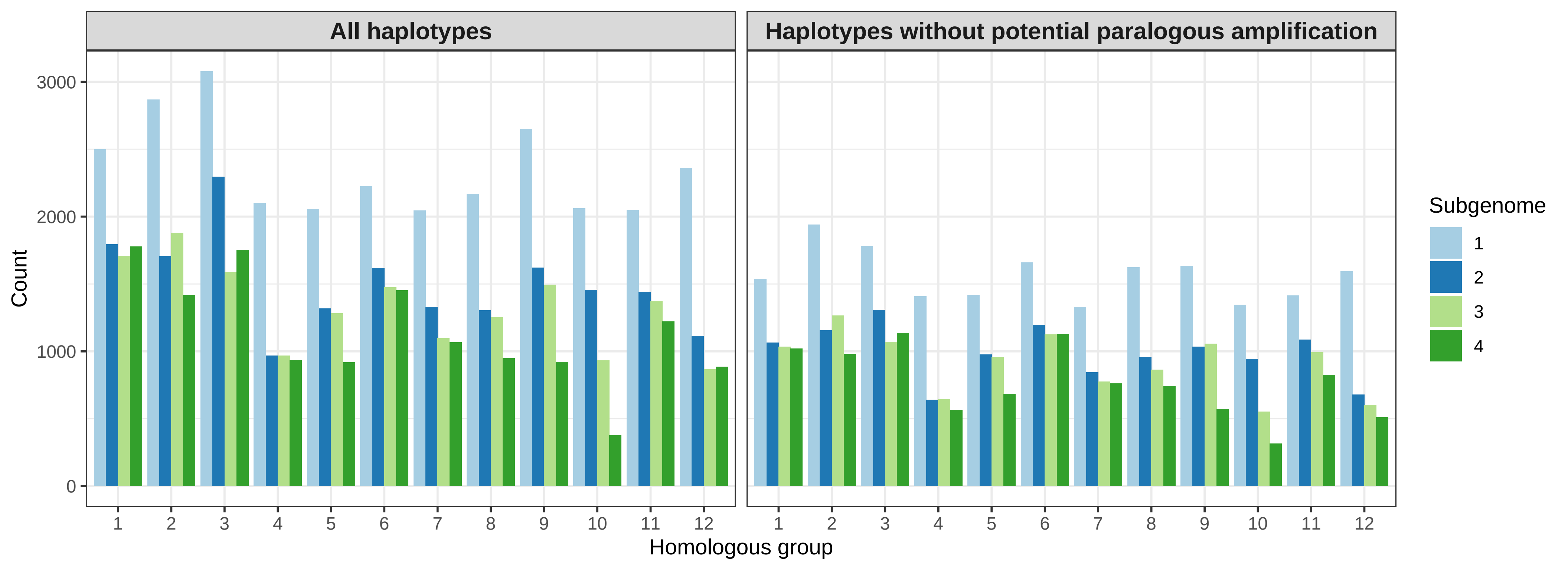

All haplotypes
Haplotypes without potential paralogous amplification
Count
3000
2000
1000
0
1 2 3 4 5 6 7 8 9 10 11 12
Homologous group
Subgenome
1
2
3
4
