# Supplementary material for: Standardized microhaplotype databases and frameworks for assessing and mining crop genetic diversity
Source: Theor Appl Genet. 2026 Aug 25;139(9):244. doi: 10.1007/s00122-026-05340-4 (PMC13506570; doi:10.1007/s00122-026-05340-4)
Supplement: Supplementary file 6 [file 122_2026_5340_MOESM8_ESM.pdf]

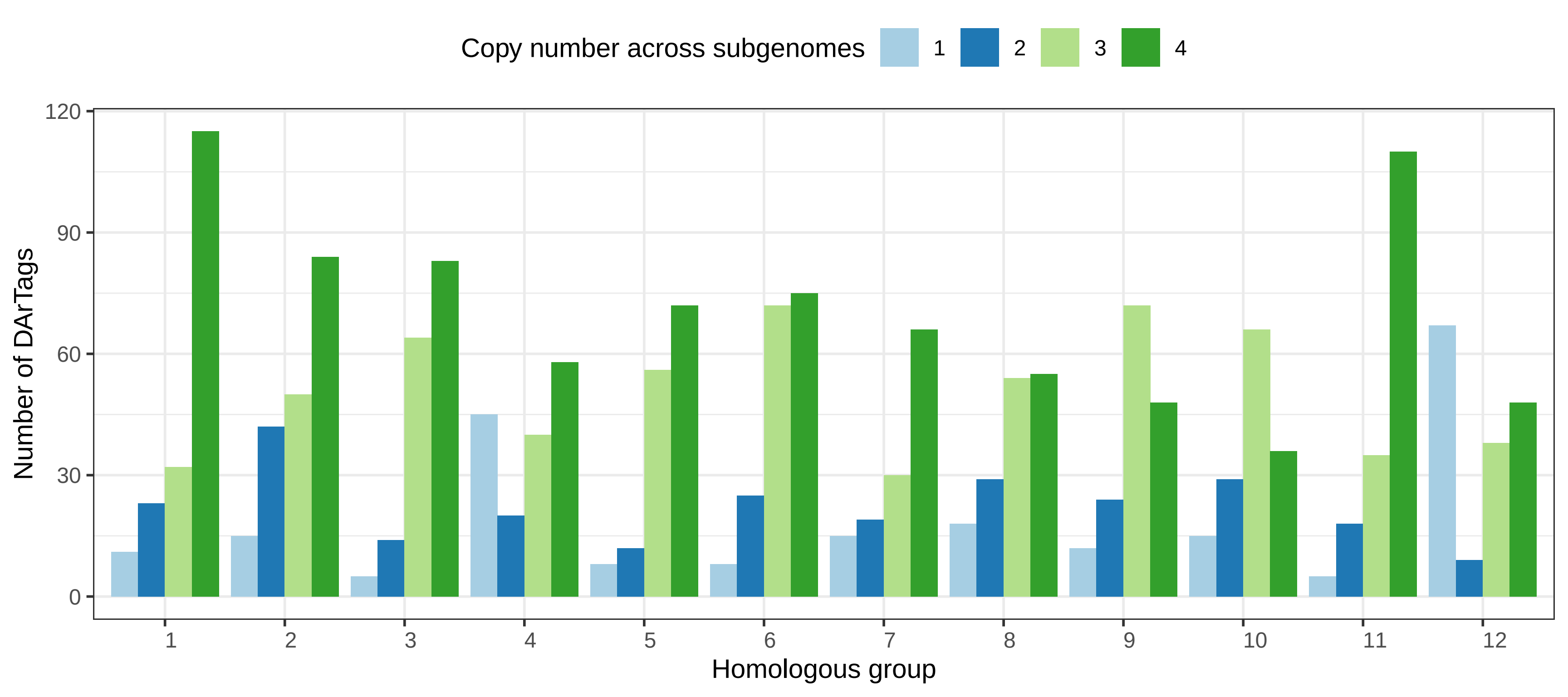
Copy number across subgenomes
1
2
3
4
Number of DArTags
120
90
60
30
0
1
2
3
4
5
6
7
8
9
10
11
12
Homologous group
